# A culturally tailored iSupport model for dementia carers: Study protocol for a hybrid type I randomised controlled trial

**DOI:** 10.1016/j.ijnsa.2026.100546

**Published:** 2026-05-02

**Authors:** Zhige Yan, Lily Xiao, Kham Tran, Rachel Milte, Claudia Meyer, Hui Chen (Rita) Chang, Shahid Ullah, Kate Laver, Ying Yu, Alison Kitson, Lee-Fay Low, Henry Brodaty, Agnieszka Chudecka, Nikolaus Rittinghausen, Areti Efthymiou, Mauricio Molinari Ulate, Yuda Turana, Kevin Kristian, Maddalena Fiordelli, Ron Sinclair, Helena Kyriazopoulos, Anne Margriet Pot

**Affiliations:** a Flinders University, Bedford Park, South Australia; b Bolton Clarke Research Institute, Australia; c Western Sydney University, Australia; d International Learning Collaborative, Australia; e University of Sydney, Australia; f The University of New South Wales, Australia; g Multicultural Aged Care, Australia; h Ethnic Communities' Council of Victoria, Australia; i Hellenic Mediterranean University, Greece; j University of Salamanca, Spain; k Universitas Katolik Indonesia Atma Jaya, Indonesia; l Università della Svizzera italiana, Switzerland; m University of Adelaide, Australia; n Multicultural Communities Council of South Australia, Australia; o Erasmus University Rotterdam, the Netherland; p University of Adelaide, Adelaide, South Australia

**Keywords:** hybrid effectiveness-implementation study, dementia, Caregivers, Community aged care, Online education, Quality of life, Virtual social support

## Abstract

**Background:**

Carers of people living with dementia from culturally and linguistically diverse backgrounds experience persistent inequities in access to, and engagement with, dementia education and support services. These inequities arise from multiple service-level and systemic barriers and contribute to poorer health and wellbeing outcomes for both carers and people with dementia. Addressing these gaps remains a priority for equitable aged care. This study protocol describes an implementation science study designed to embed a culturally tailored iSupport model within routine aged care services to reduce these inequities.

**Objective:**

This study aims to implement a culturally tailored iSupport model within routine aged care services and to evaluate its effectiveness, cost-effectiveness, and implementation strategies.

**Design:**

A pragmatic, multicentre, hybrid type 1 effectiveness–implementation randomised controlled trial.

**Setting(s):**

This study will be undertaken in community aged care settings managed by seven ethno-specific aged care organisations located in three states in Australia.

**Participants:**

Carers of people with dementia from seven culturally and linguistically diverse groups will be recruited to the study (n = 150).

**Methods:**

Eligible participants will be randomly allocated to either the intervention group or the usual care control group (n = 75 per group). Participants in the intervention group will receive a culturally tailored iSupport model comprising facilitator-enabled carer psychoeducation using the iSupport for Dementia program and case studies, peer support, needs-based service navigation, and coaching delivered in carers’ preferred language and cultural context. The intervention will be delivered over 12 months. The usual care group will be directed to Dementia Australia resources and the iSupport program website only. Primary outcomes will include quality of life for carers and for people with dementia (measured via proxy reporting). Secondary outcomes will be carers’ self-efficacy, perceived social support, and reactions to changed behaviours in care recipients; changed behaviours in care recipients and their experiences of care services (proxy measures); and cost-effectiveness using a cost–utility analysis framework. Data will be collected at baseline, 6 months, and 12 months. A process evaluation will be embedded within the trial to examine implementation strategies and influencing factors, using mainly interviews with carers and focus groups with staff at 6 and 12 months.

**Registration:**

www.anzctr.org.au ACTRN12625000587404. Registered 5 June 2025. Not yet recruiting.

**What is already known**

• Carers of people living with dementia from culturally and linguistically diverse backgrounds experience persistent inequities in access to, and engagement with, dementia education and support services.
• Embedding evidence‑based, culturally tailored education and support interventions within routine community aged care services remains challenging due to multiple barriers operating at organisational and system levels.

Alt-text: Unlabelled box dummy alt text

**hat this paper adds**

• This paper reports an innovative implementation science study using a hybrid type I effectiveness–implementation randomised controlled trial to evaluate the effectiveness, cost‑effectiveness, and implementation strategies of a culturally tailored iSupport model delivered within routine community aged care services.
• The findings will inform community aged care organisations on how to embed the culturally tailored iSupport model into routine service delivery to address inequities in dementia education and support for carers from culturally and linguistically diverse backgrounds.
• The findings will also inform policy development and funding realignment to support the scale‑up of the culturally tailored iSupport model, with the potential to reduce inequities in dementia care services and improve health and wellbeing outcomes for people living with dementia and their carers from culturally and linguistically diverse backgrounds.

Alt-text: Unlabelled box dummy alt text

## 1 Background

Dementia affects >57 million people globally and is a leading cause of death and disability in countries with ageing populations (WHO, 2021). Many high‑income countries, including Australia, Canada, the United Kingdom, and the United States, have increasingly diverse populations comprising people from culturally and linguistically diverse backgrounds (United Nations, 2024). Cultural values such as filial piety, collectivism, spirituality, and religiosity strongly influence carers to provide care for family members living with dementia at home (Lillekroken et al., 2023; Yu et al., 2018). However, government‑subsidised dementia education and support programs for carers are predominantly designed for mainstream cultural groups, with limited consideration of the perspectives, needs, and preferences of carers from culturally and linguistically diverse backgrounds (Gilbert et al., 2022; Stenberg & Hjelm, 2023). The limited availability of culturally tailored interventions contributes to health inequalities among both carers and people living with dementia from those backgrounds. Health inequalities are defined as systematic, socially produced differences in health and quality‑of‑life outcomes between population groups within a society (WHO, 2024a).

People from culturally and linguistically diverse backgrounds who live with advanced dementia are more likely to remain at home rather than enter residential aged care, influenced by cultural beliefs regarding family care for older people and the limited availability of ethno‑specific nursing homes (Stenberg & Hjelm, 2023). More than two‑thirds of people with dementia also live with multiple chronic conditions, such as hypertension, diabetes, and depression, which require substantial carer involvement in ongoing management (Temedda et al., 2025). A population‑based study indicates that carers from culturally and linguistically diverse backgrounds experience disproportionately high levels of burden and social isolation. Specifically, 67% of their care recipients had profound functional limitations and an average of 5.5 chronic conditions requiring management, compared with 56% among carers from the mainstream culture (AIHW, 2022). Furthermore, 52% of people with dementia in this population spoke English poorly or not at all, resulting in heavy reliance on family carers for activities of daily living, mobility, communication, and chronic disease management (AIHW, 2025). Behavioural and psychological changes affect up to 90% of people with dementia living in the community and are a major contributor to carers’ emotional distress, burden, and reduced quality of life (Feast et al., 2016). Despite these substantial care demands, accessible and culturally and linguistically tailored emotional support and behavioural management programs for carers remain very limited (AIHW, 2025).

Lack of preparedness for the caregiving role has been linked to low carer self‑efficacy, increased care complications, and avoidable hospital admissions (Bott et al., 2019). Language barriers limit carers in participating in publicly funded dementia education and support programs (Lillekroken et al., 2023; Yu et al., 2024). Evidence from a population‑based study in the United States suggests that approximately 40% of dementia‑related hospitalisations may be preventable through adequate carer education and support (Anderson et al., 2020). Carers from culturally and linguistically diverse communities often experience erosion of pre‑migration social support networks, leading to social isolation and limited access to peer support opportunities (Lai et al., 2019; Yu et al., 2024). Adult children caring for parents with dementia are particularly common in culturally and linguistically diverse communities, and this group is more likely to be engaged in paid employment, increasing their vulnerability to role strain (Lillekroken et al., 2023).

### 1.1 The culturally tailored iSupport model to mitigate dementia care challenges

It is estimated that >28% of people living with dementia in Australia (>115,108) are from culturally and linguistically diverse backgrounds, and 79% of this population live at home and are supported by family carers (AIHW, 2025; DoHA, 2024). Carers from culturally and linguistically diverse backgrounds account for approximately 32% of the national dementia carer population (>107,904) and experience a disproportionately high dementia care burden (AIHW, 2025; DoHA, 2024). Despite this substantial need, dementia education and support programs tailored for culturally and linguistically diverse carers remain very limited (AIHW, 2025).

**Fig. 1:**
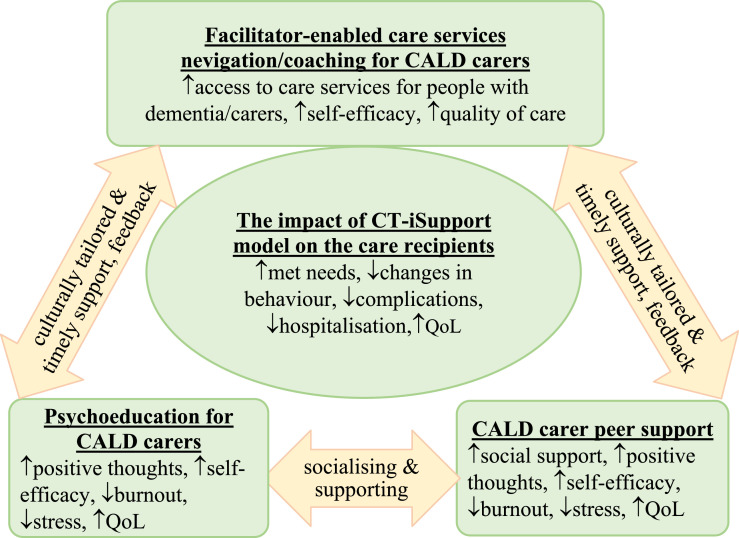
The culturally tailored iSupport model for culturally and linguistically diverse carers.

QoL: quality of life; CALD: culturally and linguistically diverse; CT-iSupport: culturally tailored iSupport model.

The culturally tailored iSupport model that will be evaluated in this study comprises three intervention components as outlined in Fig. 1. It will be delivered to carers in their preferred language and culture by trained facilitators. The three components in the model has previously been tested in a randomised controlled trial involving carers from the mainstream population in Australia (Xiao et al., 2025a). The trial showed that the iSupport model can improve carers’ health and wellbeing and reduce hospital admissions among people living with dementia. This body of evidence, together with strong national and international policy imperatives to promote health equity for people living with dementia and their carers (DoHA, 2024; WHO, 2017), provides a compelling rationale for applying an implementation science study to accelerate the translation of evidence into routine community aged care services.

#### 1.1.1 Culturally tailored psychoeducation program for carers

The psychoeducation component of the model is the iSupport for Dementia program, originally developed by the World Health Organization (WHO, 2019, 2021) and culturally adapted in Australia in eight languages: English, Greek, Bahasa Indonesia, Italian, Simplified Chinese, Spanish, Traditional Chinese, and Vietnamese. The program comprises six modules and 30 units. The modules include: Introduction to dementia; Being a carer; Caring for yourself; Providing everyday care; Dealing with changed behaviour; and My engagement in consumer‑directed care (see Supplementary File 1).

The iSupport program incorporates principles of person‑centred care, problem‑solving strategies, and self‑care through evidence‑informed case studies, enabling carers to build dementia‑specific knowledge, practical caregiving skills, and positive caregiving appraisals. These elements support carers to manage daily care tasks, prevent or respond to changed behaviours, and improve care outcomes for people living with dementia, while reducing carers’ stress and burden (Pot et al., 2019). Where appropriate, the program also introduces carers to relevant dementia care resources and multidisciplinary services to facilitate access and utilisation. Carers are encouraged to select units most relevant to their individual learning needs and incorporate them into a personalised study plan.

Consultations with key stakeholders prior to this study identified a lack of culturally tailored case studies to support carers in managing changed behaviours, chronic conditions (e.g. diabetes and hypertension), late‑stage dementia, and palliative care within an Australian sociocultural context (Xiao et al., 2026a). Stakeholders also expressed a strong preference for short videos that simulate real‑world care scenarios and could be used for self‑coaching or facilitator‑led coaching to support achievement of carers’ learning goals (Xiao et al., 2026a). To address the identified limitations, a book entitled “Culturally tailored case studies for dementia carers” with videos and podcasts was co‑designed and co-produced with stakeholders to align with the 6-module iSupport program. The case studies address: (1) caring for a person with memory loss; (2) supporting carers at the time of dementia diagnosis and beyond; (3) enabling culturally tailored self‑care for carers; (4) supporting carers in late‑stage dementia care; (5) enabling carers to support loved ones experiencing changed behaviours; and (6) enabling carers to access appropriate care services. Collectively, these comprise 56 short case scenarios presented through 54 videos and 2 podcasts (see Supplementary File 1).

#### 1.1.2 Carer peer support

Preparing staff from aged care organisations to act as facilitators of carer support groups is an effective strategy to reduce social isolation and promote peer support among carers (Xiao et al., 2026b; Yu et al., 2024). Online peer support enables carers to reinforce positive caregiving appraisals, observe role modelling, enhance self‑efficacy, and reduce caregiving‑related stress (Etxeberria et al., 2021). Findings from our feasibility study demonstrated that facilitator‑led, monthly online peer support meetings of one hour’s duration, combined with weekly text‑based interactions via social media platforms such as WhatsApp or WeChat in small groups (5–10 carers per group), were acceptable to carers (Xiao et al., 2025b).

#### 1.1.3 Needs-based care service navigation and coaching support

Trained personnel–led care service navigation for carers can address barriers within health and aged care systems, facilitate timely access to services for care recipients, prevent care crises, and reduce avoidable hospital admissions and emergency department utilisation (Kallmyer et al., 2023). Carers from culturally and linguistically diverse backgrounds have expressed a strong preference for facilitators who share their cultural and language background to support navigation of care services and overcome language barriers (Xiao et al., 2026a; Yu et al., 2024).

Carers also desire to contact facilitators for coaching support to develop problem‑solving skills when managing chronic conditions, complications, and changed behaviours affecting their care recipients (Xiao et al., 2026a). Guided by a health coaching framework (Wolever et al., 2013), coaching within the psychoeducation component of this study is defined as facilitator‑enabled processes that include learning needs assessment, goal setting, and active engagement in dementia care education through the iSupport program and the “Culturally tailored case studies” and/or related videos. To operationalise this approach, we co-developed a coaching tool for carers (Supplementary File 2) and a coaching tool for facilitators (Supplementary File 3) prior to this study.

## 2 The study

### 2.1 Aims

The aims of this study are to implement a culturally tailored iSupport model within routine aged care services and to evaluate its effectiveness, cost-effectiveness, and implementation strategies.

### 2.2 Hypotheses

#### 2.2.1 Primary hypotheses

Compared to carers allocated to the usual care group, 1) carers receiving the culturally tailored iSupport model will report (a) at least a 5-point higher mean score on the mental component of the 12-Item Short-Form Health Survey and (b) an improved mean score on the physical component of the survey; 2) their care recipients will report an improved mean score on the Quality of Life in Alzheimer’s Disease-Proxy at 12-month follow-up.

#### 2.2.2 Secondary hypotheses

Compared to those allocated to the usual care group, 1) carers in the intervention group will report (a) an improved mean score on the Caregiving Self-Efficacy Scale and (b) an improved mean score on the Quality of Social Support Scale; 2) their care recipients will report (via proxy ratings) (a) a reduced mean score on the Revised Memory and Behaviour Problem Checklist and (b) fewer unplanned hospital admissions, less emergency department uses and less use of permanent residential aged care at 12 months; and, (c) better quality care experiences via proxy ratings; and 3) provision of the culturally tailored iSupport model will be cost-effective compared to usual care.

### 2.3 Research questions for the process evaluation

We will explore the two research questions in the process evaluation: 1) what are the strategies that enable aged care organisations, staff and carers of people with dementia to embed the iSupport model in real care settings; and 2) what are the barriers that impede stakeholders from embedding the iSupport model in real care settings?

### 2.4 Methods

#### 2.4.1 Design

**Fig. 2:**
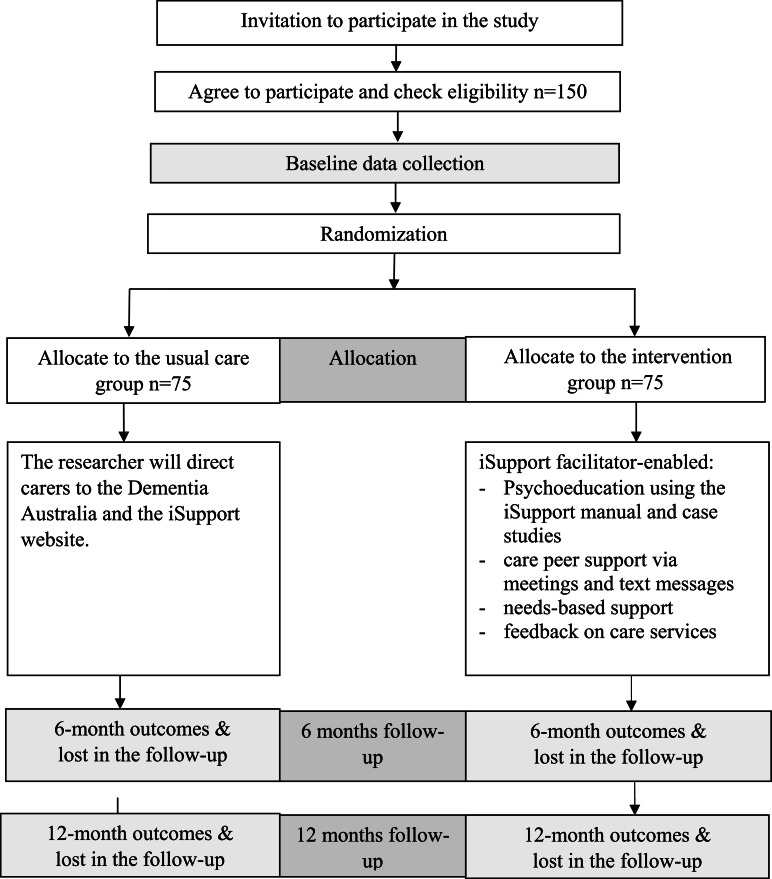
CONSORT flow chart.

A pragmatic, multicentre hybrid type I effectiveness–implementation randomised controlled trial will be conducted. This design enables the concurrent evaluation of the effectiveness of the culturally tailored iSupport model and the implementation strategies under real‑world conditions. A hybrid type I design was selected in preference to other hybrid designs because there is currently limited evidence to guide the implementation of the culturally tailored iSupport model within aged care organisations. The intervention will be delivered over a 12‑month period. Fig. 2 present the CONSORT flow chart.

A process evaluation will examine contextual factors influencing implementation, implementation fidelity, and the mechanisms through which the intervention produces change (Moore et al., 2015). The trial was registered with the Australian New Zealand Clinical Trials Registry on 5 June 2025 (ACTRN12625000587404). Further information is available in the enclosed “Information about trial registration data”.

#### 2.4.2 Study setting, participants and sample size

This study will be undertaken in community aged care settings managed by seven ethno‑specific aged care organisations located in three states in Australia. The estimated minimal sample size for carers in the study is 100 with 50 carers in the intervention group and 50 in the usual care group. This sample size is based on a primary outcome of the mental component of the 12-Item Short-Form Health Survey and an assumption of a 0.57 effect size reported in a previous trial using a multicomponent intervention for carers of people with dementia (Berwig et al., 2017), with an 80% power to detect a difference between means of 4.93 with a pooled standard deviation of 8.63 in the study. However, considering a 50% attrition rate in a 12-month trial in our previous project, we will need to recruit 150 carers to the trial or 75 carers in the tailored iSupport group and the usual iSupport group respectively.

##### 2.4.2.1 Inclusion and exclusion criteria for carers

Inclusion criteria for carers: 1) Carers speak one of these languages at home: Italian, Greek, Mandarin, Cantonese, Vietnamese, Bahasa or Spanish; 2) Carers are aged 18 years or over; 3) Carers provide care for older people (aged greater than or equal to 65) living with dementia from a culturally and linguistically diverse background at least twice a week. If the care recipients have not been formally diagnosed with dementia but meet cognitive impairment using the ‘AD8 Dementia Screening Interview’ with score equal or greater than 2 (Galvin et al., 2005). Exclusion criteria: 1) Carers have self-reported terminal health conditions that may significantly affect their ability to participate in the study; and 2) Carers are involved in other studies.

##### 2.4.2.2 Inclusion and exclusion criteria for staff

Staff will be invited to participate in the process evaluation if they meet these criteria: 1) providing direct care services to people with dementia from one of the seven language groups; 2) having dementia care experience for at least 3 years. Staff will be excluded from the study if they are agency staff.

#### 2.4.3 Randomisation

Following completion of baseline data collection, carers will be randomly allocated to either the intervention group or the usual care group. Block randomisation will be used to ensure balanced allocation to each treatment group within each recruitment site. Stratification will be incorporated into the block randomisation to ensure comparable distribution of spouse versus non‑spouse carers and similar stages of dementia among care recipients across the two groups. Randomisation will be conducted using R software version 4.3.2 by a statistician on the research team who will be blinded to participant identities and will not be involved in outcome assessment or data analysis. Group assignments will be put in sequentially numbered, opaque and sealed envelopes and opened only after each participant has completed baseline data collection.

#### 2.4.4 Interventions

##### 2.4.4.1 The intervention group

Participants in this group will receive the culturally tailored iSupport model, comprising facilitator-enabled four intervention components over a 12‑month period.

###### 2.4.4.1.1 Component 1: A psychoeducation program

First, trained facilitators will engage carers in a learning needs assessment using an adapted version of the Risk Appraisal Measure (Czaja et al., 2009). This assessment incorporates recommendations for relevant learning units from the iSupport program and unfolding case studies drawn from the “Culturally tailored case studies for dementia carers”. Based on the assessment, carers will select the recommended learning units and case studies to develop an individualised study plan and complete these at their own pace. Carers will be encouraged to reassess their learning needs and modify their study plans throughout the 12‑month intervention period in response to changes in their care recipients’ circumstances. The expected time commitment for the psychoeducation program is approximately 10–20 min per week. Engagement with the educational content and completion of study plans will be monitored through an intervention audit. All psychoeducation resources will be made available in carers’ preferred language and delivery format, including web‑based learning, e‑books, or printed hard‑copy materials.

###### 2.4.4.1.2 Component 2: Facilitator‑enabled carer peer support

The facilitator will assign carers to peer support groups of 5–10 participants and will facilitate monthly online meetings via platforms such as WhatsApp, WeChat, or Microsoft Teams. Each meeting will last approximately 45–60 min. A standardised meeting agenda will be used to guide discussions, with a focus on carers’ experiences of the psychoeducation component, including learning processes, challenges, and outcomes. Meetings will be recorded and made available to carers within the same group for later review, as needed. Between meetings, facilitators will encourage carers to engage in weekly group interactions through text messaging, sharing resources, or informal discussion via WhatsApp, WeChat, or other agreed social platforms to strengthen social connectedness and peer support.

###### 2.4.4.1.3 Component 3: Needs‑based support

Carers will be encouraged to contact their facilitator when their care recipients experience changes in health status, dementia symptoms, or transitions in care type or care setting. Facilitators will support carers to navigate, access, and utilise appropriate health and aged care services and community resources in a timely manner to address emerging needs and prevent care crises.

Facilitators will also engage carers in structured coaching activities and provide individual coaching sessions based on carers’ needs. Coaching may be delivered online or in person, according to the carer’s preference. Facilitators will continue to support carers until identified needs have been adequately addressed. Initial coaching sessions will typically last 45–60 min, with follow‑up sessions lasting approximately 5–10 min. Both carers and facilitators will be encouraged to document coaching activities using the coaching tools.

###### 2.4.4.1.4 Component 4: Carer feedback on care services

Facilitators will encourage carers to provide feedback on their experiences with care services during monthly peer support group meetings or through individual meetings with facilitators. Carers’ feedback will be discussed within participating organisations’ quality improvement meetings to inform service refinement and the development of more culturally tailored dementia care services for people living with dementia and their carers. Facilitators will also communicate organisational responses and quality improvement actions back to carers, closing the feedback loop.

##### 2.4.4.2 The usual care group

A site-specific researcher will direct carers in this group to Dementia Australia and iSupport program website for dementia care resources including the online iSupport manual in their preferred language using a flyer.

#### 2.4.5 Outcome evaluation

The scheduled time points for assessing the outcomes are outlined in Table 1 and detailed in the following sections. All instruments used in the outcome measures have been validated in English versions and have been translated and validated in the seven languages. Carer participants can choose their preferred language version in data collection.

**Table 1:** Time schedule of enrolment, interventions and assessment for participants.

	STUDY PERIOD													
	Enrolment	Allocation	Post-allocation											Close-out
Time point	-t_0_	t0 (baseline)	t_1_ (1 m)	t_2_ (2 m)	t_3_ (3 m)	t_4_ (4 m)	t_5_ (5 m)	t_6_ (6 m)	t_7_ (7 m)	t_8_ (8 m)	t_9_ (9 m)	t_10_ (10 m)	t_11_ (11 m)	t_12_ (12 m)
ENROLMENT														
Eligibility screen	X													
Informed consent	X													
Allocation		X												
INTERVENTINOS														
Psychoeducation														
Care support group														
Individualised support														
Feedback on services														
ASSESSMENT														
Demographic information		X												
Intervention effectiveness														
*Caregiver Qol: SF-12*		X						X						X
*Qol for care recipients: Qol-AD-Proxy*		X						X						X
*Caregiver self-efficacy: 8-item RSCSE*		X						X						X
*Caregiver social support: COPE Index-QS*		X						X						X
*Dementia related symptoms: RMBPC*		X						X						X
*Quality of care experience: QCE*		X						X						X
Intervention cost-effectiveness														
*Health service use*	X	X												X
*Resource usage: RUD*		X	X	X	X	X	X	X	X	X	X	X	X	X
*The incremental costs and gains in QALYs for caregivers and care recipients*		X						X						X

Allocation after baseline data collection; QoL: Quality of Life; SF-12: 12-Item Short-Form Health Survey; QOL-AD-Proxy: Quality of Life in Alzheimer’s Disease-Proxy; 8-item RSCSE: 8-item Caregiving Self-efficacy scale; COPE: Index-QS Carers of Older People in Europe Index-Quality of Social Support; RMBPC: Revised Memory and Behaviour Problem Checklist; QCE: Quality of care experience; Health service use: linked administrative health data from Data Linkage Services in Australia; RUD: Resource Utilization in Dementia Lite Questionnaire which will measure unplanned hospital admissions, emergency department use and the use of permanent residential aged care for care recipients; QALYs: Quality adjusted life years.

##### 2.4.5.1 Demographic information

Demographic information about caregivers and their care recipients will be collected at baseline only by a site-specific bilingual and bicultural researcher.

##### 2.4.5.2 Intervention effectiveness

We will collect data using a questionnaire survey with carer participants at baseline, 6 months and 12 months post-initiation of the intervention to measure intervention effectiveness. Participants can choose an online or hardcopy survey in their preferred language.

###### 2.4.5.2.1 Primary outcome measures

Carers’ quality of life will be assessed using the 12‑Item Short Form Health Survey (Ware et al., 1996). The survey comprises 12 items that measure two summary domains: mental health–related quality of life and physical health–related quality of life, with higher scores indicating better quality of life. Care recipients’ quality of life will be measured using the proxy‑reported Quality of Life in Alzheimer’s Disease scale (Logsdon et al., 2002). Scale consists of 13 items, with higher scores reflecting better perceived quality of life.

###### 2.4.5.2.2 Secondary measures

Secondary outcomes will include: (1) carer self‑efficacy, assessed using the 8‑item Caregiving Self‑Efficacy Scale (Ritter et al., 2022), with higher scores indicating greater self‑efficacy; (2) perceived quality of support, measured using a 5‑item support scale (Moholt et al., 2018), where higher scores reflect better support; (3) memory and behavioural problems of care recipients, assessed using the 24‑item Revised Memory and Behaviour Problem Checklist (Teri et al., 1992), with lower scores indicating fewer problems and reduced associated carer distress; and (4) quality of the care experience, measured using a 6‑item care‑proxy rating scale (Jemere et al., 2025), with higher scores representing a more positive care experience. In addition, healthcare utilisation outcomes, including hospital admissions, emergency department presentations, and permanent residential aged care placement, will be measured.

##### 2.4.5.3 Intervention cost-effectiveness

An economic evaluation will be conducted alongside the clinical trial using a cost-utility analysis framework, using quality-adjusted life years gained over the follow-up period from the perspective of carers in the intervention group, compared to those in the usual care group as our primary measure of benefit. Resources associated with the development and implementation of the intervention will be collected and costed according to established best practice guidelines (Drummond et al., 2015). Responses to the 12‑Item Short Form Health Survey across the follow-up period will be converted into health state utilities for the calculation of quality-adjusted life years for carers using the Short-Form 6-Dimension preference-based scoring algorithm developed by Brazier et al. (2017).

Costs to the health system will be measured via use of government-held administrative datasets, including primary care services and hospital and emergency department utilisation through government-held datasets for the comprehensive publicly-funded health system in Australia. We will also use the resource utilisation in dementia questionnaire (Wimo A, 1998) to measure health and social care visits of carers and people with dementia outside those provided those data sets. The questionnaire will be administered at baseline and then bi-monthly for 12 months (see Table 1). Unit costs will be derived from the primary care services, hospital finance departments and the Independent Health and Aged Care Pricing Authority.

The primary outcome of the economic evaluation will be presented as an incremental cost per quality-adjusted life year gained over the follow-up period for usual care compared with the intervention. Incremental cost-effectiveness ratios and cost-effectiveness acceptability curves will be estimated for varying threshold values. An assessment of the sensitivity of the results obtained to variation in measured resources, effectiveness and unit costs will be undertaken using appropriate one-way and multi-way sensitivity analysis.

##### 2.4.5.4 Process evaluation

The Integrated Promoting Action on Research Implementation in Health Services (i‑PARIHS) framework (Harvey & Kitson, 2016) will be applied to the process evaluation. The framework aligns with the UK Medical Research Council guidance for process evaluation of complex interventions (Moore et al., 2015). It conceptualises implementation as the interaction between four core constructs: innovation, recipients, context, and facilitation. These constructs provide a comprehensive lens to examine the factors influencing the implementation, embedment, and sustainment of the culturally tailored iSupport model, as described in the following sections.

First, the innovation construct emphasises the perceived relative advantage of an intervention over usual care, including its usability, credibility, and the extent to which its outcomes are observable in practice. In the context of this study, the culturally tailored iSupport model has been co‑designed with stakeholders, demonstrated effectiveness for carers and their care recipients, and shown acceptability in a pilot study (Xiao et al., 2025b, 2026a). Thus, the innovation characteristics of the model warrant further examination. In particular, how the iSupport is perceived in terms of its added value, usability, and fit within routine community aged care services needs to be explored when implemented at scale across a broader and more diverse population.

Second, the recipients construct emphasises the importance of developing in‑depth understanding of the individuals and groups involved in implementation, including their cultural values, language use, motivation, knowledge, skills, and available resources relevant to the intervention. Recipients in this study include carers from culturally and linguistically diverse backgrounds, iSupport facilitators and staff. Exploring the experiences of these recipient groups in engaging with the iSupport model is therefore essential to inform the refinement of evidence‑based implementation strategies.

Third, the context construct highlights the influence of local, organisational, and broader health and aged care system contexts on implementation processes and outcomes. Accordingly, this study will explore contextual factors shaping the iSupport model implementation, including policies, procedures, resources, staffing, education and training, workflows, and other organisational conditions that may enable or constrain staff engagement and commitment to embedding the iSupport model within routine practice.

Fourth, facilitation is conceptualised as an active and enabling ingredient for successful implementation and the achievement of desired outcomes. This construct focuses on the purposeful mobilisation of facilitation roles, skills, and strategies to assess and respond to the innovation, recipient characteristics, and contextual factors throughout the implementation process. Consistent with this framework, we previously co‑developed and pilot‑tested clearly defined facilitator roles, responsibilities, and a structured training program for iSupport facilitators who were staff members nominated by participating aged care organisations to deliver the intervention within their local service contexts (Xiao et al, 2025b). In the current study, iSupport facilitators will receive training, and their facilitation processes and implementation outcomes will be systematically monitored.

The process evaluation plan is summarised in Table 2 and described below. A mixed‑methods design will be employed, incorporating a descriptive qualitative approach using individual interviews and/or focus groups. Participants will include carers in the intervention group, iSupport facilitators, site leaders, and staff to capture diverse perspectives on the implementation and acceptability of the iSupport model. In addition, facilitators’ monthly implementation reports will be collected to identify site‑specific implementation challenges and strategies. Survey data will also be collected from carers in the intervention group to assess their satisfaction with resources and support provided during the study.

**Table 2:** A process evaluation plan informed by the i-PARIHS framework.

Elements	Explanation	Data collection methods
**The innovation**	The advantages, benefits and usability of the culturally tailored iSupport model from stakeholders’ perspectives.	Interviews or focus groups will be conducted with CALD carers, site leaders, facilitators, and staff involved in the culturally tailored iSupport model at 6 and 12 months.
**The recipient**	Cultural values, language use, motivation, knowledge, skills and resources among CALD carers and staff.	Baseline cultural and demographic information will be collected for CALD carers and staff; resources used in the study by carers, staff and organisations will be explored.
**The context**	Organisation’s policies, procedures, resources and support and aged care policies and funding arrangements that affect the culturally tailored iSupport model.	Interviews or focus groups will be conducted with CALD carers, site leaders, facilitators and staff at 6 and 12 months.
**The facilitation**	The actions/strategies, processes and outcomes in embedding the CT-iSupport; training and support for facilitators and staff; facilitators’ capabilities in implementation.	Monthly facilitators’ reports; site leaders’ comments on facilitators’ reports; interviews with site leaders, facilitators, staff and carers at 6 and 12 months.

i-PARIHS= Integrated Promoting Action on Research Implementation in Health Services; CALD= culturally and linguistically diverse.

#### 2.4.6 Data collection

##### 2.4.6.1 Data collection from carers

Site-specific researchers will collect demographic information at the baseline. They will also support carers to complete a self-administered survey at baseline, 6 months and 12 months post-initiation of the intervention. In addition, up to 30 carers in the intervention group will be invited to an interview at 6 months and 12 months post-initiation of the intervention.

##### 2.4.6.2 Data collection from staff

Up to 30 staff involved in the delivery of the iSupport model will be invited to a focus group or an interview at 6 months and 12 months post-initiation of the intervention.

##### 2.4.6.3 Data collection from the participating organisations

Data will be collected to examine organisational responses to the intervention innovation and contextual factors influencing implementation through multiple sources. First, facilitators responsible for delivering the iSupport model will document their activities and perceived outcomes using a structured portfolio. These portfolios will be reviewed monthly by site leaders to support implementation monitoring. Second, de‑identified Risk Appraisal Measure forms completed by carers will be submitted to the research team for analysis. Third, de‑identified coaching records completed by both facilitators and carers will be submitted to the project team on a monthly basis throughout the 12‑month trial period to capture the intensity, content, and responsiveness of coaching support. In addition, three monthly carer peer support meeting transcripts will be randomly selected from each peer support group for qualitative analysis to explore carers’ experiences and engagement with the peer support component.

#### 2.4.7 Data analysis

##### 2.4.7.1 Intervention effectiveness

Data analysis will be led by a statistician who will be blinded to group allocation. All analyses will be conducted according to the intention‑to‑treat principle, with participants analysed in their assigned groups. Multivariable linear mixed‑effects models will be used to examine differences in primary and secondary outcomes between groups over time. Given the repeated measurements for each participant, mixed‑effects models will account for both fixed and random effects within the hierarchical data structure. Fixed effects will include group, time, and group‑by‑time interaction terms, while random effects will model individual‑level variability in intercepts to account for within‑participant correlation. Models will be adjusted for baseline values of the outcome variables. Maximum likelihood estimation will be used to assess between‑group differences and changes over time.

Univariate mixed‑effects models will be fitted initially, followed by multivariate models incorporating variables identified as clinically relevant or statistically significant in univariate analyses to control for potential confounding. Model building will involve sequential addition and removal of covariates, with changes in model fit evaluated using log‑likelihood statistics to determine the final multivariate model. All statistical tests will be two‑sided, with statistical significance set at p < 0.05. Analyses will be performed using Stata version 16.1 and R version 4.2.2.

##### 2.4.7.2 Intervention cost-effectiveness

A cost-utility analysis will be conducted from the perspective of people living with dementia and their carers, comparing the intervention to usual care. The primary outcome will be the quality-adjusted life years gained over the follow-up period. A health economist will perform the analysis following established guidelines (Drummond et al., 2015). Resources used in developing and implementing the intervention will be identified and valued. Health state utilities for carers will be derived from 12‑Item Short Form Health Survey responses using the Short-Form 6-Dimension scoring algorithm (Brazier et al., 2017). Similarly, Quality of Life in Alzheimer’s Disease scale will be converted into utilities using the algorithm (Comans et al., 2020).

Healthcare utilisation data for people with dementia and their carers will be sourced from linked administrative datasets for the 12 months before and after recruitment, subject to participant consent. The Resource Utilisation in Dementia questionnaire (Wimo A, 1998) will capture additional health and social care visits not covered by Medical Benefits Schedule. Changes in service use and costs between the study arms will be calculated by comparing pre- and post-baseline levels. Unit costs will be obtained from the hospital finance departments, and the Independent Hospital Pricing Authority. The cost of residential aged care will be calculated based on basic daily and accommodation fees, apportioned by the length of stay. A person-level analysis will determine mean costs and quality-adjusted life years for both the control and intervention groups. Incremental cost-effectiveness ratios and cost-effectiveness acceptability curves will be estimated across a range of willingness-to-pay thresholds. The sensitivity of the results to variations in key parameters will be assessed using one-way and multi-way sensitivity analyses (Brazier et al., 2017; Drummond et al., 2015). Finally, a budget impact analysis will extrapolate the trial-based costs and outcomes to the population level (Mauskopf et al., 2007).

##### 2.4.7.3 Process evaluation data analysis

The qualitative data will be transcribed verbatim for analysis. We will enter the transcripts into a computer-assisted qualitative data analysis program, NVivo13, for data management to facilitate coding. Thematic analysis (Nowell et al., 2017) will be applied to data from focus groups, interviews and qualitative records. Descriptive data analysis will be applied to quantitative survey data.

#### 2.4.8 Fidelity of intervention

Multiple strategies have been established to ensure intervention fidelity throughout the study. First, a Steering Committee comprising members of the project team and representatives from partner organisations will be established to oversee all study processes and outcomes. The Committee will meet monthly to monitor study progress and ensure methodological rigour is maintained. Second, monthly fidelity audits will be conducted against the trial protocol to ensure consistency in intervention delivery, participant engagement and satisfaction, and implementation quality across all study sites. Third, audit findings will be reported and reviewed during monthly trial quality‑control meetings involving facilitators and researchers, where implementation challenges and potential solutions will be collaboratively discussed and addressed. Fourth, all researchers and facilitators will complete a comprehensive training program developed by the project team, consisting of five 1‑hour online training sessions, four self‑guided learning modules, and monthly 1‑hour meetings focused on intervention‑specific activities. Participants will also be provided with the culturally tailored iSupport model Implementation Manuals, which standardise intervention delivery procedures and research protocols across sites. Fifth, facilitators will systematically document key activities, implementation processes, and performance in a Facilitator’s Portfolio, which will be submitted monthly for audit and review to support ongoing fidelity monitoring and quality improvement.

#### 2.4.9 Ethical considerations

This study received ethics approval from the Flinders University Human Research Ethics Committee in August 2025 (reference number: HEG8271). Eligible participants will be recruited from seven partner organisations. Recruitment will occur in two stages. First, staff participants will be recruited through flyers and email invitations distributed within participating organisations. Staff members who meet the inclusion criteria and express interest in the study will be invited to contact the research team by returning a response sheet or by email or SMS.

Second, staff at each of the seven study sites will identify potential carers from culturally and linguistically diverse backgrounds through their organisational databases, based on the study’s inclusion and exclusion criteria. Invitations to participate, including detailed study information provided in carers’ preferred languages, will be sent to eligible carers. Interested carers will be asked to indicate their willingness to participate by contacting a bilingual researcher via return of a response sheet, email, or SMS. A researcher will then meet with interested carers to assess eligibility, explain the study procedures, and obtain written informed consent. In addition, informed consent will be obtained from people living with dementia, or third‑party consent will be obtained where appropriate, in accordance with ethical guidelines. Following consent, the researcher will conduct a cognitive assessment to determine the stage of dementia and collect demographic information from carers and care recipients.

## 3 Discussion

To our knowledge, this study is the first effectiveness–implementation randomised controlled trial to evaluate a culturally tailored dementia care model for carers from culturally and linguistically diverse backgrounds, delivered in partnership with aged care organisations. Embedding the culturally tailored iSupport model within the aged care system has the potential to represent a paradigm shift by accelerating the translation of evidence into practice and establishing a scalable, equity‑oriented care model to address longstanding health inequalities experienced by people living with dementia and their family carers from culturally and linguistically diverse backgrounds (Adsul et al., 2024; Purnell et al., 2016).

By design, the culturally tailored iSupport model constitutes a multicomponent intervention to address the complex needs of carers and care recipients (Cheng et al., 2020). Evidence from a systematic review and meta‑analyses indicates that multicomponent interventions achieve larger effect sizes than single-component approaches in supporting carers of people living with dementia (Cheng et al., 2020). In addition, the iSupport model aligns with stakeholders’ expectations for coordinated, one‑stop‑shop education and support for carers and their care recipients (Steiner et al., 2020), and is consistent with integrated care principles that emphasise person‑ and family‑centred service delivery across care settings, irrespective of location (WHO, 2024b). Despite these strengths, challenges related to embedding, scaling up, and sustaining the iSupport model within routine community aged care services are anticipated and are discussed in the following sections.

### 3.1 Carers’ factors in embedding the iSupport model

Previous studies have shown that carers from culturally and linguistically diverse backgrounds readily embraced the iSupport model, largely because of the limited availability of culturally relevant education and support programs within mainstream care systems (Xiao et al., 2026a; Yu et al., 2024). A hybrid effectiveness–implementation feasibility study of the iSupport model with Chinese‑Australian carers further demonstrated high acceptability, evidenced by strong retention rates and high levels of satisfaction with facilitator‑provided support (Xiao et al., 2025b). However, the reach of the culturally tailored iSupport model in the pilot study was relatively low (17%). Limited time due to caregiving commitments and low levels of digital literacy were identified as key barriers to participation.

Several enabling strategies were found to address these barriers, including offering hybrid delivery modes (online and face‑to‑face), providing hard‑copy versions of the iSupport manual, and appointing bilingual and bicultural facilitators as a single, trusted point of contact for needs‑based support (Xiao et al, 2026a; Yu et al., 2024). In addition, previous iSupport trials conducted in other countries have reported high attrition rates and low levels of participant engagement, which may partly explain the lack of observed intervention effectiveness in some studies (Baruah et al., 2021; Windle et al., 2025). Evidence suggests that strategies such as motivating, encouraging, and empowering carers; tailoring content, formats, and scheduling to carers’ preferences; and facilitating experiential learning and coaching activities can enhance engagement and sustained participation in dementia support interventions (Chan et al., 2024). These strategies will be explored in this study.

### 3.2 Aged care staff’s factors in embedding the iSupport model

As in other high‑income countries, the majority of the aged care workforce in Australia comprises unlicensed care workers who often have limited formal training and skills in dementia care (Dow et al., 2024). Chronic workforce challenges, including low staffing levels, burnout, and high staff turnover, further constrain the capacity of aged care services to embed and sustain evidence‑based interventions. Education and training are widely recognised as effective strategies for preparing facilitators to deliver evidence‑based interventions in complex care settings (Harvey et al., 2023). However, prior studies indicate that facilitators often enter implementation roles with varying levels of experience and competence. Consequently, creating experiential learning environments is critical to support facilitators to learn from more experienced peers, access ongoing peer support, and reflect on their practice through regular facilitator meetings and needs‑based mentoring from implementation experts (Harvey et al., 2023). The process evaluation embedded within this study will enable a detailed examination of facilitators’ and staff members’ experiences, the organisational and contextual factors influencing their engagement with and commitment to implementing the iSupport model.

### 3.3 Aged care organisations’ factors in embedding the iSupport model

The aged care sector is widely recognised as a resource‑constrained setting for translating evidence‑based dementia care interventions into routine practice, due to limited funding, high workloads among frontline staff, and a shortage of registered nurses and other health professionals to lead intervention delivery (Pace et al., 2024). In the Australian context, aged care organisations do not receive government funding to provide education and support directly to family carers. Instead, such support is primarily funded through national organisations, including Dementia Australia and the Carer Gateway (Carer Gateway, 2026; Dementia Australia, 2026). Dementia Australia delivers dementia‑specific education and support for carers of people living with dementia, while the Carer Gateway provides more generic education and support for carers across diverse care contexts, without a specific dementia focus. However, education and support programs offered by these nationally funded services are predominantly delivered in English, limiting access and utilisation among carers with limited English proficiency (Yu et al., 2024). As a result, carers from culturally and linguistically diverse backgrounds commonly seek information and assistance from ethno‑specific organisations, which are perceived as more culturally accessible and responsive (Yu et al., 2024). In addition, ethno‑specific aged care organisations play a critical role in supporting carers to navigate and access services for people living with dementia through government‑funded initiatives such as the Care Finder program (My Aged Care, 2026).

Ethno‑specific aged care organisations are well positioned to implement the culturally tailored iSupport model because of their established trust and close relationships with culturally and linguistically diverse communities. However, our pilot study identified important challenges to implementation and sustainment. Staff participation in the facilitator training program designed to support iSupport model embedment was low (36%), and the long‑term sustainability of the model remained uncertain due to the absence of dedicated funding to support the facilitator role (Xiao et al., 2025b). In contrast, most carers (72%) indicated a willingness to contribute financially to the iSupport model through their co‑funded home care budgets, reflecting their perceived value of the model and its relevance to their caregiving needs (Yu et al., 2024).

Previous research suggests that several organisational strategies may help address these implementation barriers, including providing protected time for staff to participate in education and training, adopting micro‑learning approaches to mitigate time constraints, involving staff in intervention decision‑making, and supporting site leaders and facilitators to develop leadership capability to drive organisational change (Pace et al., 2024). These research findings underscore the importance of examining organisational contextual factors through process evaluation to inform feasible and sustainable implementation strategies for the culturally tailored iSupport model within ethno‑specific aged care services.

### 3.4 Strengths and limitations of the work

A key strength of this study lies in its systematic approach to a paradigm shift in the aged care system to address inequalities in supporting carers. Building on the prior effectiveness trial of the iSupport model for mainstream carers, this study will advance the evidence base by moving into an effectiveness–implementation phase to support the embedment and sustainment of the culturally tailored iSupport model for carers from culturally and linguistically diverse backgrounds. Extensive stakeholder consultations and a pilot hybrid effectiveness–implementation trial conducted prior to this study established the feasibility and acceptability of the culturally tailored iSupport model and strengthened partnerships with seven ethno‑specific aged care organisations. The hybrid study design further enables the concurrent evaluation of intervention effectiveness and implementation strategies, thereby enhancing the relevance and applicability of findings to real‑world community aged care settings.

Several limitations should also be acknowledged. As a pragmatic, open‑label, single‑blinded randomised controlled trial, the study is subject to potential bias because carers and facilitators are aware of group allocation. In addition, the primary data sources for the process evaluation may be susceptible to social desirability bias. Finally, the inclusion of carers from only seven language groups, due to resource constraints, limits the generalisability of findings and introduces the possibility of sampling bias. These limitations will be carefully considered in the interpretation of study results.

## 4 Conclusion

This study protocol outlines a hybrid type I effectiveness–implementation randomised controlled trial designed to evaluate the effectiveness, implementation strategies, and cost‑effectiveness of the culturally tailored iSupport model for carers of people living with dementia from seven language groups in Australia. This study is expected to generate critical evidence on how to embed and sustain an evidence‑based, culturally responsive care model within dementia and aged care systems to address health inequalities experienced by people living with dementia and their carers from culturally and linguistically diverse communities. Findings from this study, including health economic evidence, will inform policy development and funding realignment to support broader implementation. Such changes have the potential to enable ethno‑specific aged care organisations to deliver the iSupport model at scale, building on their established capacity to provide culturally tailored care for older people living with dementia and their longstanding partnerships with carers from culturally and linguistically diverse communities.

## Declaration of generative AI and AI-assisted technologies in the manuscript preparation process

During the preparation of this work the authors used Microsoft Copilot to assist with grammar checking and minor language refinement. After using this tool, the authors reviewed and edited the content as needed and take full responsibility for the content of the published article.

## Funding statement

This project is funded by the 2022 National Health and Medical Research Council Targeted Call Research: Cultural, ethnic and linguistic diversity in dementia research (Grant ID: 2024551). The sponsor had no role in the design of this study, and will not have any role during its execution, analyses, interpretation of the data, or decision to submit results.

## Ethics approval statement

This study was approved by Flinders University Human Research Ethics Committee in August 2025 (reference number: HEG8271).

## Clinical trial registration

The trial is registered on the Australian New Zealand Clinical Trials Registry website (ACTRN12625000587404).

## CRediT authorship contribution statement

**Zhige Yan:** Writing – review & editing, Writing – original draft, Investigation. **Lily Xiao:** Writing – review & editing, Writing – original draft, Supervision, Project administration, Methodology, Investigation, Funding acquisition, Conceptualization. **Kham Tran:** Writing – review & editing, Project administration, Investigation, Funding acquisition. **Rachel Milte:** Writing – review & editing, Methodology, Investigation, Funding acquisition. **Claudia Meyer:** Writing – review & editing, Investigation, Funding acquisition. **Hui Chen (Rita) Chang:** Writing – review & editing, Project administration, Investigation, Funding acquisition. **Shahid Ullah:** Writing – review & editing, Supervision, Methodology, Investigation, Funding acquisition, Formal analysis. **Kate Laver:** Writing – review & editing, Writing – original draft, Methodology, Investigation, Funding acquisition. **Ying Yu:** Writing – review & editing, Investigation, Funding acquisition. **Alison Kitson:** Writing – review & editing, Investigation, Funding acquisition. **Lee-Fay Low:** Writing – review & editing, Funding acquisition. **Henry Brodaty:** Writing – review & editing, Funding acquisition. **Agnieszka Chudecka:** Writing – review & editing, Funding acquisition. **Nikolaus Rittinghausen:** Writing – review & editing, Funding acquisition. **Areti Efthymiou:** Writing – review & editing, Funding acquisition. **Mauricio Molinari Ulate:** Writing – review & editing, Funding acquisition. **Yuda Turana:** Writing – review & editing. **Kevin Kristian:** Writing – review & editing. **Maddalena Fiordelli:** Writing – review & editing, Funding acquisition. **Ron Sinclair:** Writing – review & editing, Funding acquisition. **Helena Kyriazopoulos:** Writing – review & editing, Funding acquisition. **Anne Margriet Pot:** Writing – review & editing, Funding acquisition.

## Declaration of competing interest

The authors declare the following financial interests/personal relationships which may be considered as potential competing interests:

Lily Xiao reports financial support was provided by National Health and Medical Research Council. If there are other authors, they declare that they have no known competing financial interests or personal relationships that could have appeared to influence the work reported in this paper.

## Appendix Supplementary materials

Image, application 1

Image, application 2

Image, application 3

Image, application 4

Image, application 5

Image, application 6
