# Supplementary material for: A culturally tailored iSupport model for dementia carers: Study protocol for a hybrid type I randomised controlled trial
Source: Int J Nurs Stud Adv. 2026 May 2;10:100546. doi: 10.1016/j.ijnsa.2026.100546 (PMC13136732; doi:10.1016/j.ijnsa.2026.100546)
Supplement: Supplementary file 1 [file mmc1.pdf]

Australian Government
National Health and Medical Research Council

NHMRC

Targeted Call for Research: Cultural, ethnic and linguistic diversity in dementia research 2022 Guidelines

**Opening date:**	21 September 2022
**Closing date and time:**	17.00 AEDT on 16 November 2022
**Commonwealth policy entity:**	National Health and Medical Research Council (NHMRC)
**Administering entity**	NHMRC
**Enquiries:**	Applicants requiring further assistance are to direct enquiries to their Administering Institution's Research Administration Officer. Research Administration Officers can contact NHMRC's Research Help Centre for further advice:<br>Phone: 1800 500 983 (+61 2 6217 9451 for international callers)<br>Email: help@nhmrc.gov.au<br>NHMRC will not respond to any enquiries submitted after 13:00 AEDT on 16 November 2022.<br>Note: NHMRC's Research Help Centre aims to provide a reply to all requests for general assistance within two working days. This timeframe may be longer during peak periods or for more detailed requests for assistance.
**Date guidelines released:**	21 September 2022
**Type of grant opportunity:**	Targeted competitive

BUILDING
A HEALTHY
AUSTRALIA

Contents

1. Targeted Calls for Research: Cultural, ethnic and linguistic diversity in dementia research 2022 processes 5

1.1 Introduction 6

1.1.1. About NHMRC 6

2. About the grant program 6

2.1 Key changes 9

3. Grant amount and grant period 9

3.1 Grants available 9

3.2 Grant period 10

4. Eligibility criteria 10

4.1 Who is eligible to apply for a grant? 10

4.1.1 Chief Investigators and Associate Investigators 10

4.2 Multiple applications/grants 11

4.3 Exclusion of applications 11

5. What the grant money can be used for 11

5.1 Eligible grant activities and expenditure 11

5.1.1 Salary support 11

5.2 Duplicate funding 12

5.3 What the grant money cannot be used for 12

6. The assessment criteria 12

6.1 Health research involving Aboriginal and Torres Strait Islander People 13

7. How to apply 14

7.1 Overview and timing of grant opportunity processes 14

7.2 Application Extensions 15

7.3 Minimum data requirements 15

7.4 Application requirements 16

7.5 Attachments to the application 16

7.6 Consumer and community involvement 16

7.7 Certification and submission 17

7.7.1 CIA certification 17

7.7.2 Certification from other Chief Investigators (CIB-CIJ) and Associate Investigators 17

7.7.3 Administering Institution certification 18

7.8 Retracted publications 18

7.9 Withdrawal of applications 19

7.10 Questions during the application process 19

8. The grant selection process 19

8.1 Assessment of grant applications 19

8.1.1 Who will assess applications? 19

8.1.2 TCR: Cultural, ethnic and linguistic diversity in dementia research 2022 grant opportunity assessment process 20

8.2 Who will approve grants? 20

9. Notification of application outcomes 20

10. Successful grant applications 20

10.1 Information required from grantees 21

10.2 Obligations and approvals 21

10.3 NHMRC Funding Agreement 21

10.3.1 Responsible and ethical conduct of research 21

10.4 NHMRC policies 21

10.5 Payments 21

10.6 Suspension of grants 22

10.7 Tax implications 22

11. Announcement of grants 22

12. How we monitor your grant activity 22

12.1 Variations 22

12.2 Reporting 22

12.2.1 Financial reports 22

12.2.2 Non-financial reports 23

12.3 Evaluation of the TCR scheme 23

12.4 Open Access Policy 23

13. Probity 23

13.1 Complaints process 23

13.2 Conflicts of Interest 23

13.3 Privacy: confidentiality and protection of personal information 24

13.4 Freedom of information 24

14. Glossary 25

Appendix A. TCR: Cultural, ethnic and linguistic diversity in dementia research 2022 Category Descriptors 28

Appendix B. TCR: Cultural, ethnic and linguistic diversity in dementia research Guide to Applicants 35

1. Preparing an application 35

2. Application Requirements 35

2.1 Use of gender-neutral language 35
2.2 Minimum Data Requirements 36
3. Key Changes 36
4. 'My Profile' Requirements 37
4.1 About My Profile 37
4.2 Personal information 37
4.3 Academic Information 37
4.4 Peer Review Information 38
4.5 Unavailability Calendar 38
4.6 Contributions to NHMRC 38
5. 'My Profile' Requirements specific to TCR: Cultural, ethnic and linguistic diversity in dementia research 2022 grant opportunity 38
5.1 Career Disruptions (within the last 5 years) 39
5.2 Relative to Opportunity (within the last 5 years) 39
5.3 Community Engagements (within the last five years) 40
5.4 Translation into Policy/Practice (within the last five years) 40
6. Application Form Requirements 41
6.1 Creating an application 41
6.2 Application details 41
6.3 Participating Institutions 43
6.4 Research Classification 43
6.5 Research Team 44
6.6 Ethics 45
6.7 Grant Proposal 45
6.8 Third Party Research Facilities 50
6.9 Direct Research Costs 50
7. Certifying your application 52
8. Checklist for applicants 52

1. Targeted Calls for Research: Cultural, ethnic and linguistic diversity in dementia research 2022 processes

NHMRC's Targeted Calls for Research (TCR) scheme is designed to achieve Australian Government objectives
The TCR scheme is a component of the Portfolio Budget Statements
Program 1.1: Health and Medical Research, which contributes to Outcome 1: Improved health and medical knowledge.

↓

The grant opportunity opens
NHMRC publishes the grant opportunity guidelines on GrantConnect.

↓

Applicants complete and submit a grant application
Applicants must complete the application form and address all of the eligibility criteria to be considered for a grant.

↓

Applications are verified and assessed
Applications are verified against eligibility criteria and applicants are notified if not eligible. Peer reviewers assess applications against the assessment criteria including an overall consideration of value with money.

↓

Grant decisions are made
NHMRC's CEO seeks approval of funding recommendations from the Minister for Health.

↓

NHMRC notifies applicants of the outcome

↓

Applicant's Administering Institution signs the NHMRC Grant Schedule(s) setting out the grant activity

↓

Delivery of grant
Grantees undertake the grant activity as set out in the schedule to the Funding Agreement.
NHMRC manages the grant through the relevant Administering Institution.

↓

Evaluation of the TCR scheme
NHMRC undertakes periodic evaluations of the performance and administration of its funding schemes to determine strengths and to identify where improvements can be made.

1.1 Introduction

These grant opportunity guidelines (guidelines) contain information for the TCR: Cultural, ethnic and linguistic diversity in dementia research 2022 grant opportunity.

Applicants must read these guidelines before filling out an application.

This document sets out:

- the purpose of the grant scheme/grant opportunity
- the eligibility and assessment criteria
- how grant applications are considered and selected
- how grantees are notified and receive grant payments
- how grants will be monitored and evaluated
- responsibilities and expectations in relation to the opportunity.

GrantConnect (www.grants.gov.au) is the authoritative source of information on this grant opportunity. Any alterations or addenda to these guidelines will be published on GrantConnect.

The TCR: Cultural, ethnic and linguistic diversity in dementia research 2022 grant opportunity will be undertaken in accordance with the *Commonwealth Grants Rules and Guidelines 2017* (CGRGs), available from the Department of Finance website.

Commonwealth funding for this grant opportunity, including where future or additional funding opportunities are indicated, is subject to the relevant Commonwealth Government funding policy and priorities at the time of notification and accordingly may be subject to change. This may affect the funding available, and timing of such, provided under this grant opportunity. Any such changes will be notified as soon as possible.

NHMRC recognises the impacts of the COVID-19 pandemic on Australia's health and medical research community. NHMRC's Relative to Opportunity Policy specifies that circumstances associated with the pandemic and other calamities are considered, where applicable, in assessment of an applicant's track record. In their application, applicants may outline the interruption and impact on their research productivity.

1.1.1. About NHMRC

NHMRC is the Australian Government's key entity for managing investment in, and the integrity of, health and medical research. NHMRC works with stakeholders to plan and design the grant program in accordance with the *National Health and Medical Research Council Act 1992* (NHMRC Act) and the CGRGs.

NHMRC awards grants through several research funding schemes to advance health and medical knowledge and to improve the health status of all Australians. NHMRC invests in the highest quality research and researchers, as determined through peer review, across the four pillars of health and medical research: basic science, clinical medicine and science, public health and health services research.

2. About the grant program

A TCR is a one-time solicitation for grant applications to address a specific health issue and stimulate research in areas of national importance where there is a significant knowledge gap. A TCR specifies the scope and objectives of the research to be proposed, application requirements and procedures, and the review criteria to be applied in the evaluation of applications submitted in response to the TCR.

In alignment with NHMRC's Corporate Plan, the objective of the TCR scheme is to fund targeted research that responds to unmet or emerging health needs and reflects national, state and territory, and consumer and community priorities.

The TCR scheme:

- complements NHMRC's other funding schemes by funding priority research in defined areas of need and when urgent research needs emerge
- stimulates or greatly advances research in a particular area of health and medical science that will benefit the health of Australians.

Funding for the program will be provided from the NHMRC Medical Research Endowment Account (MREA), which is underpinned by section 51 of the NHMRC Act.

The **TCR: Cultural, ethnic and linguistic diversity in dementia research 2022** grant opportunity is a component of the Portfolio Budget Statements (PBS) Program 1.1: Health and Medical Research, which contributes to Outcome 1: Improved health and medical knowledge.

It is estimated that 487,500 people are currently living with dementia and 1.6 million Australians are involved in the care of someone living with dementia. Dementia is a serious chronic health condition that can present in people as young as 30 but is more commonly diagnosed in those over 65 years of age. It is the second leading cause of death for Australians and the leading cause of death for women.[1,2] In June 2020, 16% of the Australian population was over 65 and projections suggest that by 2066 this will rise to between 21% and 23% of the total population.[3,4]

Current evidence suggests that Australian dementia research frequently underrepresents people from diverse cultural, ethnic or linguistic backgrounds and that relevant data on ethnicity are not routinely gathered or reported.[1] As approximately one-third of Australia's population aged over 65 are from diverse cultural, ethnic or linguistic background, this represents a major research gap as they potentially represent a significant proportion of people living with dementia, or likely to be in the future as the population becomes increasingly multicultural.[5]

In 2015, NHMRC established the NHMRC National Institute for Dementia Research (NNIDR) to target and coordinate the $200 million national dementia research effort through the Boosting Dementia Research Initiative (BDRI). While all BDRI funding opportunities have now been awarded, NHMRC remains committed to dementia research offering funding opportunities across selected NHMRC grant schemes.

In conjunction with the National Ageing Research Institute, the NNIDR developed the *Culturally And Linguistically Diverse (CALD) Dementia Research Action Plan* which identified research priorities to increase and promote the inclusion of all members of the Australian community in dementia research and improve dementia health and care equity. Development of the plan

[1] Australian Bureau of Statistics (2020). Causes of Death, Australia, 2019 (cat. No. 3303.0)

[2] Australian Institute of Health and Welfare (2020). Dementia Snapshot. https://www.aihw.gov.au/reports/australias-health/dementia

[3] Australian Bureau of Statistics (2020). Older Australians Demographic Profile. https://www.aihw.gov.au/reports/older-people/older-australians/contents/demographic-profile

[4] Australian Bureau of Statistics (2018). Older Australians Demographic Profile. https://www.aihw.gov.au/reports/older-people/older-australians/contents/demographic-profile

[5] Australian Bureau of Statistics (2017). Census of Population and Housing: Reflecting Australia – Stories from the Census 2016 [Online] Available: https://www.abs.gov.au/ausstats/abs@.nsf/Lookup/by%20Subject/2071.0~2016~Main%20Features~About%20this%20Release~9999 [Accessed 28/05/2019].

included extensive consultation with consumers, carers, advocates, policy makers and prominent dementia researchers.

In addition, the Federation of Ethnic Communities Councils of Australia published *If we don't count it… It doesn't count! Towards consistent national data collection and reporting on cultural, ethnic and linguistic diversity* which identified the inadequacies in the collection and reporting of cultural, ethnic and linguistic diversity data. The current body of dementia research does not reflect the ethnic, linguistic and cultural diversity of the Australian population. Hence, people from cultural, ethnic and linguistic diverse backgrounds may receive inequitable dementia care as there is less evidence to guide optimal clinical and service decisions.

Significant disparities in health and care outcomes between cultural, ethnic and linguistic diverse Australians and the general population have been noted including: lower rates of specialist referral and use of antidementia medication, higher rates of delayed diagnosis and medication discontinuation, and difficulty finding culturally relevant aged care services. The exclusion of people from cultural, ethnic or linguistic diverse backgrounds from dementia research limits its ability to inform sound, evidence-based approaches to meet dementia-related challenges for all members of the community. A compounding factor is that, as the symptoms of dementia worsen, individuals will lose proficiency in second languages and revert to their original language, highlighting the need for supportive treatment options and resources for non-English speaking members of the community.

The purpose of the **TCR: Cultural, ethnic and linguistic diversity in dementia research 2022 grant opportunity** is to stimulate research that includes culturally, ethnically and linguistically diverse individuals and is conducted in a culturally safe and appropriate manner. This will contribute to best practice approaches to dementia research conducted in Australia.

The objectives of the TCR are to:

- promote the inclusion and participation of, and data design/collection from, individuals from cultural, ethnic and linguistic diverse backgrounds in dementia research to ensure that future research outputs are representative of all members of the Australian community living with dementia
- understand the prevalence and aetiology of dementia in people from diverse cultural, ethnic and linguistic backgrounds and identify any unique characteristics, attitudes, barriers or risk factors, with a view to increasing participation in prevention and early intervention strategies
- develop dementia screening and assessment tools that are appropriate for people from all cultural, ethnic and linguistic backgrounds
- develop evidence-based models of dementia care that are culturally specific and accommodate the preferences and needs of patients, their families and carers, and inform health professionals and care staff on how culture influences engagement with the Australian health system.

The outcomes of the research will increase the quality and availability of evidence that is representative of all members of the Australian community with dementia. This research outcomes will better inform future research, policy, programs and health services and lead to the development of clinical pathways for the diagnosis, early intervention, treatment and management of dementia that are appropriate for people from diverse cultural, ethnic and linguistic backgrounds, as well as providing support for their families and carers. It will also enhance the design and relevance of future dementia research by increased engagement and recruitment of people from cultural, ethnic and linguistic diverse backgrounds.

Research proposals should consider the following guiding principles for culturally, ethnically and linguistically inclusive dementia research:

- Co-design and partnership approaches should involve culturally, ethnically, and linguistically diverse communities and other stakeholders over the lifespan of research projects.
- Collection, analysis, and reporting in experimental and epidemiological studies should include cultural, ethnic and linguistic diversity variables.
- Innovations or approaches should be accessible to everyone in the Australian community, regardless of culture or language.
- Researchers should work to develop and support partnerships with the health sector, care providers, training organisations, ageing and ethnic peak-bodies, and culturally, ethnically and linguistically diverse communities to ensure rapid translation of evidence into practice.
- The many unique culturally, ethnically and linguistically diverse communities within Australia should be acknowledged and any actions or interventions should be relevant to the respective needs and nuances of the target communities.

2.1 Key changes

Applicants need to note the following changes for the TCR: Cultural, ethnic and linguistic diversity in dementia research 2022 grant opportunity from previous TCRs:

- Applications submitted to this TCR will be assessed by a peer review panel comprised of two groups of peer reviewers:
    - Health and medical researchers
    - Consumer and community representatives (CCRs).

  Previous TCRs have included CCRs as members on peer review panels, providing scoring members of the panel with qualitative feedback on how well applicant teams have involved consumer and community perspectives in their research proposals. For this TCR, CCRs will provide a score that contributes to 20% of the overall assessment score. There are dedicated assessment criteria and category descriptors for each peer reviewer group. For more detail, see Section 6 and Appendix A.

- Chief Investigators (CIA-CIJ) are required to nominate up to 10 of their best publications from the past 10 years, taking into account career disruption, within the Sapphire application form. This information will be used by the health and medical research peer reviewers to assess track record.

- NHMRC's *Open Access Policy* (Section 12.4) has been updated to require all peer-reviewed publications arising from NHMRC-funded research to be:
    - made available immediately upon publication, removing the 12-month embargo period
    - published with the use of an open licence, which means publications can be used and shared widely.

3. Grant amount and grant period

3.1 Grants available

The provisional funding allocation for the TCR: Cultural, ethnic and linguistic diversity in dementia research 2022 is $3 million. Following peer review, the top ranked applicants will be funded from within the available funding allocation. NHMRC's Research Committee annually reviews and recommends indicative budget amounts to be awarded across individual funding schemes.

3.2 Grant period

A TCR grant can be requested for between 1 and 5 years depending on the proposal.

4. Eligibility criteria

Applications will only be accepted from NHMRC Administering Institutions. A list of NHMRC Administering Institutions is available on NHMRC's website.

The Chief Investigator A (CIA) and Administering Institution must ensure applications and grants meet all eligibility requirements as set out in these guidelines. Applications that do not meet these eligibility requirements may be ineligible and may be excluded from further consideration.

Where an eligibility ruling is being considered, NHMRC may request further information in order to assess whether the eligibility requirement has been met.

Decisions are made based on current policies and considerations specific to this grant opportunity. Decisions made in relation to previous grant opportunities or other NHMRC funding schemes will not be regarded as precedents and will not be considered when assessing compliance with the requirements of this grant opportunity.

Administering Institutions will be notified in writing of ineligible applications and are responsible for advising applicants of the decision.

Grant offers may be withdrawn if eligibility criteria are not met. Action may also be taken over the life of a grant if eligibility criteria to continue holding a grant are not met.

NHMRC staff will not make eligibility rulings before the submission of minimum data (at the earliest) or a full application.

4.1 Who is eligible to apply for a grant?

4.1.1 Chief Investigators and Associate Investigators

The maximum number of CIs allowed on a TCR: Cultural, ethnic and linguistic diversity in dementia research 2022 grant opportunity application is 10.

Chief Investigator 'A'

At the time of acceptance and for the duration of a grant the CIA must be an Australian or New Zealand citizen, or a permanent resident of Australia, or have an appropriate work visa in place. The CIA must also be based in Australia for at least 80% of the funding period.

Chief Investigators

The role and contribution of each CI must be described in the grant application. PhD students may be named as CIs where the PhD student is critical for the successful completion of the proposed research. CIs are expected to remain on the grant activity for the duration of the grant, unless a variation is approved by NHMRC in accordance with the *NHMRC Grantee Variations Policy*. NHMRC will only approve a change in CIs in exceptional circumstances and a variation request must not be used as a means to meet NHMRC eligibility requirements.

Associate Investigators

An Associate Investigator (AI) is defined as an investigator who provides some intellectual and/or practical input into the research and whose participation may warrant inclusion of their name on any outputs (e.g. publications).

There is no restriction on who may be named as an AI on an application. However, a maximum number of 10 can be entered in Sapphire.

4.2 Multiple applications/grants

Limits apply to the number of NHMRC grants that a CI may concurrently apply for and/or hold.

Applicants can only apply as CIA on one application to any particular TCR. Applicants can apply as a CI on a maximum of two applications to any particular TCR. Applicants who wish to apply as a CI on a second application to the same TCR can do so as CIB-CIJ, ensuring that they are listed as CIA on one application only.

Note: Applications to TCRs are not capped relative to Investigator, Synergy and Ideas Grants and will not impact a CI's ability to apply to these schemes. Further information can be found on the NHMRC website.

4.3 Exclusion of applications

An application may be excluded from further consideration if NHMRC identifies that:

- it contravenes an eligibility rule or other requirement as set out in these guidelines
- it, or any CI named on the application, contravenes an applicable law or code, or
- it is inconsistent with the objectives of the NHMRC Act and/or the purposes of the MREA.

An application will be excluded if any CI named on the application is the subject of a decision by NHMRC's CEO or delegate that any application they make to NHMRC, for specified funding schemes, will be excluded from consideration for a period of time, whether or not they otherwise meet the eligibility requirements. Such decisions will generally reflect consequential action taken by NHMRC in response to findings of a serious breach of the *Australian Code for the Responsible Conduct of Research* (the Code) (including a finding of research misconduct, where this term is used) or a Probity Event. See the Code for a definition of 'research misconduct' and the *NHMRC Research Integrity and Misconduct Policy* available from NHMRC's website.

Such exclusion may take place at any time following CIA and Administering Institution certification of the application.

If a decision is made to exclude an application from further consideration, NHMRC will provide its decision and the reason(s) for the decision to the Administering Institution's Research Administration Officer (RAO). The Administering Institution's RAO is responsible for advising applicants of the decision. Decisions to exclude an application may be reviewable by NHMRC's Commissioner of Complaints.

5. What the grant money can be used for

5.1 Eligible grant activities and expenditure

Funding provided by NHMRC for a grant activity must be spent on costs directly incurred in that grant activity that satisfy the principles and requirements outlined in the *Direct Research Costs Guidelines* on the NHMRC website.

5.1.1 Salary support

TCR grants are not normally intended to provide salary support for CIs. However, if applicants are seeking CI salaries, justification on how the proposed budget is directly associated with achieving the outcomes of the research must be provided and will be considered during peer review.

CIs, including the CIA, can draw a salary from the TCR: Cultural, ethnic and linguistic diversity in dementia research 2022 grant if they are based in Australia for at least 80% of the funding period. CIs based overseas are not able to draw a salary but the grant can be used to provide salary

support for research support staff based overseas (see section 5.2). Requested salaries must be based on Personnel Support Packages (PSPs) outlined on the NHMRC website.

Applicants can receive up to 100% salary across all NHMRC grants held. Multiple partial salaries can be drawn up to 100%, if allowed in the guidelines for the respective grant opportunities.

Associate Investigators cannot draw a salary from any TCR grants.

5.2 Duplicate funding

NHMRC may compare the research proposed in grant applications with grants previously funded, currently funded and funded by other agencies (e.g. Australian Research Council or Department of Health) and published research. NHMRC will not fund research that it considers duplicates research previously or currently being funded.

Where NHMRC believes that an applicant has submitted similar research proposals to NHMRC and has been successful with more than one application, the applicant may be required to provide NHMRC with a written report clearly identifying the difference between the research aims of the research activities. If NHMRC subsequently does not consider the research activities to be sufficiently different, the applicant will be required to decline or relinquish one of the grants.

5.3 What the grant money cannot be used for

Funding provided by NHMRC for a Research Activity may only be expended on Direct Research Costs (DRCs) as described in the NHMRC Direct Research Costs Guidelines on the NHMRC website.

The TCR: Cultural, ethnic and linguistic diversity in dementia research 2022 grant funding cannot be used for the following items:

- Severance and termination payments and extended leave payments (leave entitlements accrued on non-NHMRC Research Activities)
- Equipment costing more than $80,000 and equipment service and repair costs
- Personal computers, related peripherals or software needed for communicating, writing and undertaking simple analyses
- Fringe Benefits Tax (FBT)
- Land, buildings and fixtures
- Travel, if not directly related to the research objectives of the Research Activity.

When investigators apply for research funding, it is not possible to predict where and how knowledge translation and knowledge transfer of their work will occur (because the research is yet to be undertaken). Thus, the costs of the following items are not to be included as DRCs in grant application budgets:

- Publication and open access costs
- Conference attendance costs.

However, over the grant lifetime, funds can be used to support reasonable costs associated with the aforementioned items that are the result of the Research Activity and are in accordance with the DRC principles.

6. The assessment criteria

Applications for the TCR: Cultural, ethnic and linguistic diversity in dementia research 2022 grant opportunity are assessed by a panel of peers against the assessment criteria listed below and the category descriptors at Appendix A. The panel is comprised of two groups of peer reviewers:

- Health and medical researchers
- Consumer and community representatives.

Health and medical researchers (80% of overall assessment score)

Assessment criteria

Scientific quality and relevance to the objectives and expected outcomes of the proposed research (50%) - the clarity and potential application of the hypotheses or research objectives, the strengths and weaknesses of the study design, feasibility and sustainability.

Team capacity and record of achievement of the team in areas/disciplines relevant to this TCR – relative to opportunity (30%) - the value of the team members' past research, research activities and linkages, and other relevant achievements, relative to opportunity. Assessment comprises leadership, publications, and research impact, as well as any other relevant contributions.

Record of achievement are assessed Relative to Opportunity, taking into consideration any career disruptions, where applicable.

It is recognised that Aboriginal and Torres Strait Islander applicants often make additional valuable contributions to policy development, clinical/public health leadership and/or service delivery, community activities and linkages, and are often representatives on key committees. If applicable, these contributions will be considered when assessing research output and track record.

Consumer and community representatives (20% of overall assessment score[6])

Assessment criteria

Consumer and community involvement activities - evidence that consumers and/or the community were consulted/involved in the design of the research proposal, including detail of what ongoing community/consumer involvement will occur during the research and methods for communication of results.

Support for consumer and community involvement - the research team's demonstrated experience in working with consumers and communities and the provision of governance arrangements to support involvement (i.e. consumer input to advisory committees, planned activities or specific roles for community members), as well as adequate time and resourcing to involve and support the involvement of consumer and community representatives in the research.

Relevance and research impact for consumers and community - the proposed research addresses the objectives of the call in a way that is relevant to the needs of consumers and the community and gives appropriate consideration to the needs of people from different and diverse cultural backgrounds or who have a higher disease burden or poorer health outcomes. The outcomes of research will make a difference by improving health services or health policy that will lead to improved health outcomes for individuals.

6.1 Health research involving Aboriginal and Torres Strait Islander People

As part of NHMRC's stated commitment to advancing Aboriginal and Torres Strait Islander health research, NHMRC has requirements and processes designed to ensure that Aboriginal and Torres

---

[6] The three CCR criteria are equally weighted across the overall 20% contribution.

Strait Islander health research is of the highest scientific merit and is beneficial and acceptable to Aboriginal and Torres Strait Islander peoples and communities.

Applicants proposing to undertake research which specifically relates to the health of Aboriginal and Torres Strait Islander peoples, or which includes distinct Aboriginal and Torres Strait Islander populations, biological samples or data, must refer to the following documents in formulating their proposal:

- *Road map 3: A strategic framework for improving Aboriginal and Torres Strait Islander health through research*
- *Ethical conduct in research with Aboriginal and Torres Strait Islander Peoples and communities: Guidelines for researchers and stakeholders*, and
- *Keeping Research on Track II*, which is a companion document on how the values and principles outlined in the *Ethical conduct in research with Aboriginal and Torres Strait Islander Peoples and communities: Guidelines for researchers and stakeholders* can be put into practice in research.

To qualify as Aboriginal and Torres Strait Islander health research, at least 20% of the research effort and/or capacity-building must relate to Aboriginal and Torres Strait Islander health.

Qualifying applications must address NHMRC's *Indigenous Research Excellence Criteria* as follows:

- Community engagement - the proposal demonstrates how the research and potential outcomes are a priority for Aboriginal and Torres Strait Islander communities with relevant community engagement by individuals, communities and/or organisations in conceptualisation, development and approval, data collection and management, analysis, report writing and dissemination of results.

- Benefit - the potential health benefit of the project is demonstrated by addressing an important health issue for Aboriginal and Torres Strait Islander people. This benefit can have a single focus or affect several areas, such as knowledge, finance and policy or quality of life. The benefit may be direct and immediate, or it can be indirect, gradual and considered.

- Sustainability and transferability - the proposal demonstrates how the results of the project have the potential to lead to achievable and effective contributions to health gain for Aboriginal and Torres Strait Islander people, beyond the life of the project. This may be through sustainability in the project setting and/or transferability to other settings such as evidence-based practice and/or policy. In considering this issue, the proposal needs to address the relationship between costs and benefits.

- Building capability - the proposal demonstrates how Aboriginal and Torres Strait Islander people, communities and researchers will develop relevant capabilities through partnerships and participation in the project.

Peer reviewer(s) with specific expertise in Indigenous health research will evaluate how well the application addresses the *Indigenous Research Excellence Criteria*. This evaluation will be taken into consideration in the overall assessment of the application, using the assessment criteria outlined in Section 6 (it does not alter the weighting of the assessment criteria).

7. How to apply

7.1 Overview and timing of grant opportunity processes

**21 September 2022**	Applications open in Sapphire

**5.00pm AEDT**<br>**02 November 2022**	Minimum data due in Sapphire
**5.00pm AEDT**<br>**16 November 2022**	Applications close in Sapphire
**February – April 2023**	Anticipated peer review period*
**September 2023**	Anticipated notification of outcomes*

*Date is indicative and subject to change.

Applications must be submitted electronically using Sapphire (unless otherwise advised by NHMRC).

Electronic submission requires Administering Institutions and all CIs on an application to register for an account in Sapphire. Applicants who are not registered can submit a new user request via the login page of Sapphire.

Applicants should refer to the *Sapphire Learning and Training Resources* for detailed user instructions or contact their RAO or NHMRC's Research Help Centre for further assistance.

Late applications will not be accepted.

7.2 Application Extensions

Requests for application extensions will be considered on a case-by-case basis and must be submitted by email to help@nhmrc.gov.au before the application closing date and time. Requests will only be considered for:

- unforeseen circumstances, e.g. natural calamities such as bushfires, floods or cyclones
- exceptional circumstances that affect multiple researchers, e.g. power and/or internet network outages, or
- where an applicant, or a member of their immediate family[7], is incapacitated due to an unforeseen medical emergency, such as life-threatening injury, accident or death.

Extensions will be for a maximum of seven calendar days. This is to ensure that subsequent peer review processes and approval of funding recommendations are not delayed, especially as eligibility decisions for some NHMRC schemes depend on an applicant's success with other schemes.

Requests for extensions submitted after the scheme close date and time will not be considered.

7.3 Minimum data requirements

Minimum data must be entered in Sapphire by the specified due date. Applications that fail to satisfy this requirement will not be accepted. Applicants must complete the required fields with correct information. Placeholder text such as “text”, “synopsis” or “xx” etc. is not acceptable as minimum data.

Applicants are discouraged from making any changes to minimum data fields following the minimum data deadline as NHMRC uses minimum data to identify appropriate peer reviewers to

[7] Immediate family comprises a spouse, child, parent or sibling. It includes de facto, step and adoptive relations (e.g. de facto, step or adopted children).

assess the application. Incorrect minimum data may result in less suitable peer reviewers assessing the application.

Minimum data fields for the TCR: Cultural, ethnic and linguistic diversity in dementia research 2022 grant opportunity are outlined in Appendix B: Guide to Applicants (see section 2.2, 'Minimum Data Requirements') and within Sapphire.

Failure to meet this deadline will result in the application not proceeding. RAOs are not required to certify applications for the purpose of minimum data. Applications are only to be certified once complete and ready for submission.

7.4 Application requirements

The application must contain all information necessary for assessment without the need for further written or oral explanation or reference to additional documentation. Further information on what can and cannot be included in the application is provided in the Guide to Applicants at Appendix B.

All details included must be current at the time of submission, as this information is relied on during assessment.

Applications must comply with all content and formatting requirements. Incomplete or non-compliant applications may be ineligible.

Additional requirements and guidance for each component of the application are outlined at Appendix B.

7.5 Attachments to the application

NHMRC requires the following documents with your application:

- Completed grant proposal (see Appendix B, Section 6.7)

If applicants are engaging services from third party research facilities, letters from the research facilities confirming their collaboration are required (see Appendix B, Section 6.8).

You must attach supporting documentation to the application in line with the instructions provided in Sapphire or Appendix B. Only attach requested documents. NHMRC will not consider information in attachments that it does not request.

7.6 Consumer and community involvement

The *Statement on Consumer and Community Involvement in Health and Medical Research* (the Statement) has been developed because of the important contribution consumers make to health and medical research. The Statement's purpose is to guide research institutions, researchers, consumers and community members in the active involvement of consumers and community members in all aspects of health and medical research. The Consumers Health Forum of Australia Ltd and NHMRC worked in partnership with consumers and researchers to develop the Statement.

To complement the statement, NHMRC has released a Toolkit with resources on consumer and community involvement in, and expectations of, health and medical research. Researchers are encouraged to consider the benefits of actively engaging consumers and to use this Toolkit throughout all stages of research, including the planning and preparation of grant applications, the conduct of research and the evaluation of outcomes.

Further information on The Consumers Health Forum, the Statement and the Toolkit is available on NHMRC's website. Consumer and community involvement in the proposed research will be considered against the applicable consumer and community assessment criteria (see Section 6). For further guidance on how applicants should address consumer and community involvement within research proposals, see Appendix B, Section 10.5 Grant Proposal.

7.7 Certification and submission

Once complete, applications must be electronically certified and then submitted to NHMRC through the RAO of an NHMRC Administering Institution using Sapphire.

Certification is required firstly by the CIA and then by the Administering Institution RAO by the specified due date or the application will be ineligible and excluded from further consideration.

Once submitted to NHMRC, the application is considered final and no changes can be made.

7.7.1 CIA certification

The following assurances, acknowledgements and undertakings are required of the CIA before submitting an application:

- All required information has been provided and is complete, current and correct.
- All eligibility and other application requirements have been met.
- All personnel contributing to the grant activity have familiarised themselves with the *Australian Code for the Responsible Conduct of Research*, the *National Statement on Ethical Conduct in Human Research*, the *Australian code for the care and use of animals for scientific purposes* and other relevant NHMRC policies concerning the conduct of research, and agree to conduct themselves in accordance with those policies.
- The application may be excluded from consideration if found to be in breach of any requirements, in accordance with the guidelines.

And if funded,

- The research will be carried out in strict accordance with the conditions governing NHMRC grants at the time.
- The Head of Department of the Administering Institution (and Participating Institution/s, if applicable) will ensure the appropriate facilities will be available.
- The research may be used for internal NHMRC quality evaluations/reviews.

7.7.2 Certification from other Chief Investigators (CIB-CIJ) and Associate Investigators

By accepting an invitation to participate on an application, Chief and Associate Investigators certify that, at the time of application submission, they:

- Agree to be named on the application
- Endorse application certification by the Chief Investigator A and submission for endorsement by the Administering Institution's RAO
- Have familiarised themselves with the *Australian Code for the Responsible Conduct of Research*, the *National Statement on Ethical Conduct in Human Research*, the *Australian code for the care and use of animals for scientific purposes* and other relevant NHMRC policies concerning the conduct of research, and agree to conduct themselves in accordance with those policies

- Agree to participate in the manner described in the application and to the handling of personal information contained within the application as described in the *NHMRC Privacy Policy*
- have met all eligibility and other application requirements.

7.7.3 Administering Institution certification

The following assurances, acknowledgements and undertakings are required of the Administering Institution before submitting an application:

- Reasonable efforts have been made to ensure the application is complete and correct and complies with all eligibility and other application requirements.
- CIA is an Australian or New Zealand citizen or permanent resident at the time of accepting the successful grant.
- CIA will be based in Australia for at least 80% of the funding period.
- Where the CIA is not an Australian or New Zealand citizen or permanent resident, they will have the requisite work visa in place at the time of accepting the successful grant and will be based in Australia for at least 80% of the funding period.
- The appropriate facilities and salary support will be available for the funding period.
- Approval of the grant activity by relevant institutional committees and approval bodies, particularly for ethics and biosafety, will be sought and obtained before the commencement of the research, or the parts of the research that require their approval.
- Arrangements for the management of the grant have been agreed between all institutions associated with the application.
- The application is being submitted with the full authority of, and on behalf of, the Administering Institution, noting that under section 136.1 of the *Commonwealth Criminal Code Act 1995*, it is an offence to provide false or misleading information to a Commonwealth body in an application for a benefit. This includes submission of an application by those not authorised by the Institution to submit applications for funding to NHMRC.

Administering Institutions must ensure that the RAO is authorised to certify and submit applications.

7.8 Retracted publications

If a publication relevant to an application is retracted after the application has been submitted, the applicant must promptly notify their RAO. The RAO must advise NHMRC at the earliest opportunity of the retraction by email (help@nhmrc.gov.au) with an explanation of the reasons for the retraction.

In addition, where the publication forms part of the applicant's track record, the applicant must immediately record that information in their Profile in Sapphire.

If an application is largely dependent on the results of a retracted publication, the applicant should also consider withdrawing the application. If, under these circumstances, an applicant chooses not to withdraw the application, the RAO must advise NHMRC in writing (to help@nhmrc.gov.au), clearly outlining the reasons for not withdrawing the application.

7.9 Withdrawal of applications

Applications may be withdrawn at any time by written notice from the Administering Institution's RAO to NHMRC.

An application may be 'marked for deletion' by the applicant in Sapphire before the close of the round. This authorises NHMRC to delete the application once the grant opportunity has closed. The application will not be deleted while the grant opportunity remains open for application submission.

7.10 Questions during the application process

Applicants requiring further assistance should direct enquiries to their Administering Institution's RAO.

All policy enquiries must be submitted in writing by the Administering Institution's RAO to NHMRC's Research Help Centre. Policy enquiries from applicants will be re-directed to the RAO. Frequently asked policy questions will be addressed via the scheme's Frequently Asked Questions (FAQs) document, which will be updated on GrantConnect as required and should be reviewed before submitting a query.

NHMRC's Research Help Centre details:

Phone: 1800 500 983 (+61 2 6217 9451 for international callers)

Email: help@nhmrc.gov.au

Refer to the Research Help Centre webpage for opening hours.

8. The grant selection process

8.1 Assessment of grant applications

NHMRC considers applications through a targeted competitive grant process. Applications are required to meet eligibility requirements as set out in these guidelines and are assessed against the assessment criteria (see Section 6) by independent peer reviewers.

The extent to which applications represent value with relevant money is considered as part of the broader category descriptors at Appendix A, which guide assessment of applications against the scheme's objectives and intended outcomes (Section 2), the quality of the proposed research and the track record or capability of the applicant(s).

8.1.1 Who will assess applications?

NHMRC's peer review process is designed to provide a rigorous, fair, transparent and consistent assessment of the merits of each application to ensure that only the highest quality research that provides value with money is recommended for funding.

Applicants must not seek to identify or make contact about their application with anyone who is directly engaged with its assessment, in keeping with NHMRC's principles of impartial and independent peer review. Seeking to influence the process or outcomes of peer review may constitute a breach of the *Australian Code for the Responsible Conduct of Research* and may result in the application being excluded from consideration.

8.1.2 TCR: Cultural, ethnic and linguistic diversity in dementia research 2022 grant opportunity assessment process

NHMRC will conduct peer review for this funding round in accordance with the following principles:

- Fairness. Peer review processes are fair and seen to be fair by all involved.
- Transparency. All stages of peer review are transparent.
- Independence. Peer reviewers provide independent advice. There is also independent oversight of peer review processes by independent Chairs and Observers.
- Appropriateness and balance. The experience, expertise and operation of peer reviewers are appropriate to the goals and scale of the funding vehicle.
- Research community participation. Persons holding taxpayer-funded grants should willingly make themselves available to participate in peer review processes, including mentoring of junior researchers, whenever possible.
- Confidentiality. Participants respect that confidentiality is important to the fairness and robustness of peer review.
- Impartiality. Peer review is objective and impartial, with appropriate processes in place to manage real and perceived conflicts of interest (CoI).
- Quality and excellence. NHMRC will continue to introduce evidence-based improvements into its processes to achieve the highest quality decision-making through peer review.

Peer reviewers will independently undertake an assessment of applications against the assessment criteria (see Section 6). Some applications may be discussed by peer reviewers. The overall scores from assessments will be used to produce a rank ordered list of applications, on which funding recommendations will be based.

Further information on the assessment process is on the NHMRC website.

8.2 Who will approve grants?

In accordance with paragraph 7(1)(c) of the NHMRC Act, NHMRC's CEO makes recommendations on expenditure from the MREA to the Minister with portfolio responsibility for NHMRC. The Minister, acting on the advice of the CEO, determines expenditure from the MREA (subsection 51(2) of the NHMRC Act).

9. Notification of application outcomes

NHMRC will advise applicants and their nominated Administering Institution's RAO of the outcome of the application as early as possible, following the approval of grants. Advice of outcomes may occur before the approval of grants if an application has been assessed as uncompetitive or excluded for other reasons.

NHMRC may advise applicants and their Administering Institution's RAO of the outcome under embargo. This means that the information must not be made public until the embargo is lifted. During the embargo period, applicants must not publicise the information or post comments about the grant outcomes in public domains such as social forums, websites, journals or newspapers. NHMRC's website provides further information on what can and cannot happen where information on a grant is released under embargo.

10. Successful grant applications

CIAs whose applications are approved for funding will have access to a letter of offer through Sapphire. Administering Institutions responsible for administering approved applications will also have access to the letter of offer and to the Schedule to the Funding Agreement. The Administering Institution is responsible for accepting the Schedule through the online signing/acceptance process within Sapphire.

NHMRC's CEO or delegate may withdraw or vary an offer of a grant if they consider that it is reasonably necessary to protect Commonwealth revenue.

10.1 Information required from grantees

Grantees may be required to supply additional information about their grant activity before payments commence. This will be stated in the Schedule to the Funding Agreement, relevant grant opportunity guidelines or letter of offer.

10.2 Obligations and approvals

NHMRC-funded grant activities must comply with applicable guidelines, laws and approval requirements. For further information see NHMRC's website.

Institutions applying for NHMRC funding (both Administering and Participating Institutions) must also be aware of their obligations under The *National Redress Scheme for Institutional Child Sexual Abuse – Grant Connected Policy*. Relevant institutions which have been named in an application for the Redress Scheme or named in the Royal Commission, and which have not joined the Redress Scheme, will be ineligible to receive NHMRC funding.

NOTE: NHMRC-funded research with ethics and biosafety considerations must be referred for approval to the relevant institutional committees and approval bodies.

10.3 NHMRC Funding Agreement

All grants are offered in accordance with the Funding Agreement (with any conditions specified in Schedules and these guidelines), which is a legal agreement between NHMRC and the Administering Institution. Schedule(s) are accepted by the Administering Institution electronically in accordance with the provisions of the Funding Agreement.

Details of the Funding Agreement can be found on NHMRC's website under Funding Agreement and Deeds of Agreement. A grant will not commence, nor grant funds be paid, until:

- the Funding Agreement between NHMRC and the Administering Institution is in place, and
- the appropriate Schedule to the Funding Agreement is executed in accordance with clause 2.3 of the Funding Agreement.

10.3.1 Responsible and ethical conduct of research

NHMRC expects the highest levels of research conduct and integrity to be observed in the research that it funds. Under the Funding Agreement, NHMRC funded research must be conducted in accordance with the *Australian Code for the Responsible Conduct of Research*. Further information about the Code can be found on NHMRC's website.

10.4 NHMRC policies

Under the Funding Agreement, it is the responsibility of Administering Institutions and CIs to be aware of, and comply with, all relevant legislation and policies relating to the conduct of the grant activity.

For further information see NHMRC's website.

10.5 Payments

Payments will commence once all outstanding obligations (e.g. conditions, eligibility rules or data requirements specified in the Schedule to the Funding Agreement, relevant grant opportunity guidelines or letter of offer) have been met by the CIA and the Administering Institution.

10.6 Suspension of grants

NHMRC funding may be suspended for a variety of reasons including, but not limited to, requests made by the CIA. Variations will generally only be granted if allowed in the grant opportunity guidelines and the NHMRC *Grantee Variation Policy* available on the NHMRC website.

Funding may also be suspended by NHMRC, in circumstances as set out in the Funding Agreement, including when there has been a failure to comply with a Policy or Guideline, or on the basis of a Probity Event or an investigation of an alleged breach of the *Australian Code for the Responsible Conduct of Research* (including research misconduct, where this term is used).

10.7 Tax implications

All amounts referred to in these guidelines are exclusive of GST, unless stated otherwise.

Administering Institutions are responsible for all financial and taxation matters associated with the grant.

11. Announcement of grants

Grant outcomes are publicly listed on the GrantConnect website within 21 calendar days after the date of effect as required by the CGRGs.

12. How we monitor your grant activity

12.1 Variations

A variation is a change (including a delay) to a grant. There are specific circumstances under which grantees are to report and seek approval of a variation to an NHMRC grant (including the grant activity) relative to the peer reviewed application. Requests must comply with the grant opportunity guidelines and the *NHMRC Grantee Variations Policy*. Requests to vary the terms of a grant are to be made to NHMRC via the Grantee Variation portal in Sapphire. For information on grant variations see the *NHMRC Grantee Variations Policy* available on the NHMRC website.

Note that CIs are expected to remain on the grant for the full funding period and NHMRC will only approve changes to CIs in exceptional circumstances. Before a CIA applies for a grant variation, they and the relevant RAO will need to confirm that all CIs have agreed to the variation, noting the impact that it may have on their suite of grants and their eligibility to hold/apply for other grants. Grant variations cannot be used as a means to meet NHMRC eligibility requirements.

12.2 Reporting

Administering Institutions are required to report to NHMRC on the progress of the grant and the use of grant funds. Where an institution fails to submit reports (financial or otherwise) as required, NHMRC may take action under the provisions of the Funding Agreement. Failure to report within timeframes may affect eligibility to receive future funding.

12.2.1 Financial reports

Annual financial reports are required in a form prescribed by NHMRC. At the completion of the grant or upon transfer to a new Administering Institution, a financial acquittal is also required. Refer to NHMRC's website for details of format and timing.

12.2.2 Non-financial reports

The Funding Agreement requires the CIA to prepare reports for each grant activity. Scientific reporting requirements can be found on NHMRC's website. While having outstanding obligations from previous NHMRC grants does not disqualify applicants from applying for other NHMRC grants, it is a condition of funding that outstanding obligations from previous NHMRC grants, including submission of a Final Report, have been met before acceptance of a new grant.

Information included in the Final Report may be publicly released. Use of this information may include publication on NHMRC's website, publicity (including release to the media) and the promotion of research achievements.

The Administering Institution is also required to provide NHMRC with any other report in respect of any research activity within the timeframe, in the format and containing the information requested by NHMRC. All information provided to NHMRC in reports may be used for internal reporting and reporting to government. This information may also be used by NHMRC when reviewing or evaluating funded research projects or funding schemes, or designing future schemes.

12.3 Evaluation of the TCR scheme

NHMRC undertakes periodic evaluations of the performance and administration of its grant opportunities to determine their effectiveness and to identify where improvements can be made.

12.4 Open Access Policy

All recipients of NHMRC grants must comply with all elements of NHMRC's *Open Access Policy* as a condition of funding. NHMRC's *Open Access Policy* is available on NHMRC's website.

13. Probity

13.1 Complaints process

Applicants or grantees can lodge a formal complaint about an NHMRC process related to funding via the Administering Institution's RAO and in writing to NHMRC Complaints Team at: complaints@nhmrc.gov.au. Complaints must be lodged within 28 days of the relevant NHMRC decision or action. NHMRC will provide a written response to all complaints. NHMRC will not review the merits of a funding decision but it will investigate complaints about the administrative process followed to reach a funding decision.

If applicants or grantees are dissatisfied with the response from the NHMRC Complaints Team, they can raise their concerns with the NHMRC Commissioner of Complaints (the Commissioner). Note that the Commissioner of Complaints does not undertake a merits review. Refer to NHMRC's Complaints Policy and the Commissioner of Complaints webpage for further information.

Applicants or grantees can complain to the Commonwealth Ombudsman if they do not agree with the way NHMRC has handled their complaint. The Ombudsman will not usually consider a complaint unless the matter has first been raised directly with NHMRC and, where relevant, the Commissioner of Complaints.

13.2 Conflicts of Interest

NHMRC is committed to ensuring that interests of any kind are dealt with consistently, transparently and with rigour, in accordance with sections 16A and 16B of the Public Governance, Performance and Accountability Rule 2014 (made under the subsection 29(2) of the *Public Governance, Performance and Accountability Act 2013* (PGPA Act)).

Applicants are not required to declare actual or perceived interests.

To manage any conflicts of interest with applicants, NHMRC requires peer reviewers to declare interests, actual or perceived, and sign deeds of confidentiality. Peer reviewers declare any direct or indirect, pecuniary or non-pecuniary interest, which is reviewed by NHMRC, before being granted full access to an application. Any peer reviewer who is determined by NHMRC to have a 'high' conflict of interest will not be able to participate in the review of that application.

By managing any conflict, NHMRC maintains objectivity, impartiality and integrity in the assessment of applications. Further information about the conflict of interest process is available in the TCR: Cultural, ethnic and linguistic diversity in dementia research 2022 Peer Review Guidelines.

13.3 Privacy: confidentiality and protection of personal information

NHMRC treats applicants' personal information in accordance with the Australian Privacy Principles and the *Privacy Act 1988*. The NHMRC Privacy Policy details the types of personal or sensitive information that may be collected by NHMRC and how it will be handled. Applicants need to familiarise themselves with the NHMRC Privacy Policy before providing personal information to NHMRC.

Information that is generally regarded as confidential information is application information and any other information specifically identified as such by applicants and grantees, and will be received by NHMRC on the basis of a mutual understanding of confidentiality.

NHMRC may disclose personal and/or confidential information to:

- overseas entities, Australian, State/Territory or local government agencies, organisations or individuals where necessary to assess an application or to administer a grant
- the peer review committee and other Commonwealth employees and contractors to help NHMRC manage the grant scheme effectively
- employees and contractors of NHMRC to research, assess, monitor and analyse schemes and activities
- employees and contractors of other Commonwealth agencies for relevant purposes, including government administration, research or service delivery
- other Commonwealth, State, Territory or local government agencies in reports and consultations
- NHMRC approved Administering Institutions' Research Administration Offices
- the Auditor-General, Ombudsman or Privacy Commissioner
- the responsible Minister or Parliamentary Secretary, and
- a House or a Committee of the Australian Parliament.

In addition, NHMRC will provide certain limited personal information of the Chief Investigator/s included in an application to Administering Institutions for the purpose of certification of eligibility requirements.

13.4 Freedom of information

NHMRC as a Commonwealth agency is subject to the *Freedom of Information Act 1982* and is committed to meeting the Australian Government's transparency and accountability requirements. Freedom of Information laws facilitate the general public's access to documents held by national

government agencies, including application and funding documentation relating to NHMRC researchers. This right of access is limited where documents, or parts of documents, are exempt under the provisions of the *Freedom of Information Act 1982*.

Researchers are to familiarise themselves with NHMRC's Freedom of Information procedures before submitting an application. Further information on the *Freedom of Information Act 1982*, NHMRC's Freedom of Information application process and relevant contacts can be found on the NHMRC website.

14. Glossary

Term	Definition
Aboriginal and Torres Strait Islander descent	Identification of Aboriginal and Torres Strait Islander descent follows the advice given on the AIATSIS website (https://aiatsis.gov.au/family-history/you-start/proof-aboriginality). This states that government agencies and communities usually accept three 'working criteria' as confirmation of Aboriginal or Torres Strait Islander heritage, namely:<br>• being of Aboriginal or Torres Strait Islander descent<br>• identifying as an Aboriginal or Torres Strait Islander person, and<br>• being accepted as such by the community in which you live, or formerly lived.
assessment criteria	The specified principles or standards against which applications will be judged. These criteria are used to assess the merits of proposals and, in the case of a competitive granting opportunity, to determine applicant rankings.
Commonwealth Grants Rules and Guidelines 2017 (CGRGs)	The CGRGs establish the overarching Commonwealth grants policy framework and the expectations for all non-corporate Commonwealth entities in relation to grants administration.
date of effect	This will depend on the particular grant. It can be the date on which the schedule to a grant agreement is executed or the grant is announced, whichever is later.
eligibility criteria	The principles, standards or rules that a grant applicant must meet to qualify for consideration of a grant.
final year	The final 12 calendar months of a grant.
Funding Agreement	For NHMRC MREA grants, the grant agreement is the NHMRC Funding Agreement and the Schedule to the Funding Agreement. It is available on NHMRC's website.

Term	Definition
grant	As defined in the NHMRC Funding Agreement.
grant activity	Defined as “Research Activity” in the NHMRC Funding Agreement.
grant opportunity guidelines	All the documents published on GrantConnect under the grant opportunity. Also referred to as guidelines in this document.
grant opportunity	Refers to the specific grant round or process where a Commonwealth grant is made available to potential grantees. Grant opportunities may be open or targeted, and will reflect the relevant grant selection process.
grant program	A group of one or more grant opportunities under a single entity Portfolio Budget Statement Program. This is referred to as a scheme in this document.
GrantConnect	GrantConnect is the Australian Government’s whole-of-government grants information system, which centralises the publication and reporting of Commonwealth grants in accordance with the CGRGs. It is available at www.grants.gov.au<br><br>Non-corporate Commonwealth entities (such as NHMRC) must publish grant opportunities on GrantConnect to meet the grant publishing requirements under the CGRGs.<br><br>Where information is published in more than one location, and there are inconsistencies, GrantConnect is the authoritative, auditable information source.
Grantee	An individual/organisation that has been awarded a grant. For NHMRC’s purposes, grants are awarded to the Administering Institution for the benefit of the grant recipients (however described).
Medical Research Endowment Account (MREA)	A ‘Special Account’ established under section 49 of the NHMRC Act, through which Government appropriated funds are used to pay NHMRC grants.
Medical Research Future Fund (MRFF)	The MRFF was established in 2015 by the *Medical Research Future Fund* Act 2015 (MRFF Act). Refer to the Department of Health website:<br><br>https://www.health.gov.au/initiatives-and-programs/medical-research-future-fund

Term	Definition
peer reviewers	Individuals (peers) with appropriate knowledge and expertise who review grant applications.
Portfolio Budget Statement (PBS) Program	Described within the entity's PBS, PBS programs each link to a single outcome and provide transparency for funding decisions. These high level PBS programs often comprise a number of lower level, more publicly recognised programs, some of which will be Grant Programs (schemes). A PBS Program may have more than one Grant Program (scheme) associated with it, and each of these may have one or more grant opportunities.
Probity Event	As defined in the NHMRC Funding Agreement.
Sapphire	NHMRC's electronic, secure system that allows research administrators, applicants, assessors, grant holders and NHMRC staff to manage all aspects of the granting lifecycle.
Schedule	As defined in the NHMRC Funding Agreement.
value with money	Value with money in this document refers to 'value with relevant money' which is a judgement based on the grant proposal representing an efficient, effective, economical and ethical use of public resources and determined from a variety of considerations.<br>When administering a grant opportunity, an official should consider the relevant financial and non-financial costs and benefits of each proposal including, but not limited to:<br>• the quality of the project proposal and activities;<br>• fitness for purpose of the proposal in contributing to government objectives;<br>• that the absence of a grant is likely to prevent the grantee and government's outcomes being achieved; and<br>• the potential grantee's relevant experience and performance history.

Appendix A. TCR: Cultural, ethnic and linguistic diversity in dementia research 2022 Category Descriptors

Applications submitted to this grant opportunity are assessed by two groups of peer reviewers:

- Health and medical researchers
- Consumer and community representatives.

Each group of reviewers assesses against specific weighted assessment criteria, with scores from each group combined to calculate an overall score.

Category descriptors are used as a guide to scoring an application against each of the respective assessment criteria. While the category descriptors provide peer reviewers with some benchmarks for appropriately scoring each application, **it is not essential that all descriptors relating to a given score are met.**

The category descriptors are a guide to a 'best fit' outcome. Peer reviewers will consistently refer to these category descriptors to ensure thorough, equitable and transparent assessment of applications.

Health and medical researchers (80% of overall assessment score)

Assessment criteria

Scientific quality and relevance to the objectives and expected outcomes of the proposed research (50%) - the clarity and potential application of the hypotheses or research objectives, the strengths and weaknesses of the study design, feasibility and sustainability.

Team capacity and record of achievement of the team in areas/disciplines relevant to this TCR - **relative to opportunity (30%) -** the value of the team members' past research, research activities and linkages, and other relevant achievements, relative to opportunity. Assessment comprises leadership, publications, and research impact, as well as any other relevant contributions.

Category	*Scientific quality and relevance to the objectives and expected outcomes of the TCR (50%)*	*Record of achievement of the team in areas/disciplines relevant to this TCR – relative to opportunity (30%)*
**7** *Outstanding*	The **research proposal**:<br>• is **highly relevant** to the call<br>• is **without question, highly feasible**<br>• has objectives that are **well-defined, highly coherent** and **strongly developed**<br>• has a **near flawless** design<br>• has **very comprehensive** strategies for performance measures/milestones and how grant funds and other resources will be shared, deployed, and redeployed<br>• will provide very **high quality** evidence that addresses the expected outcomes of the call<br>• is **highly competitive** with the best comparable research proposals internationally<br>• is **highly likely** to be translated into, or	The **applicant team:**<br>• has a track record that is **highly relevant** to the proposed research in depth and breadth<br>• has **all** the required skills and expertise to achieve the expected outcomes<br>• is **highly networked** with domestic and/or international alliances to exchange knowledge and skills.

	inform changes in, policy or health practice<br>• will **almost certainly** result in highly influential publications and research outputs	
**6**<br>***Excellent***	The **research proposal**:<br>• is **very relevant** to the call<br>• is **highly feasible**<br>• has objectives that are **clear**, **logical** and **well developed** that advances knowledge<br>• is **excellent** in design<br>• has **comprehensive** strategies for performance measures/milestones and how grant funds and other resources will be shared, deployed, and redeployed<br>• will provide **high quality** evidence that addresses the expected outcomes of the TCR<br>• is very **likely** to be competitive with strong comparable research proposals internationally<br>• is very **likely** to be translated into, or inform changes in, policy or health practice<br>• is **very likely** to result in highly influential publications	The **applicant team:**<br>• has a track record that is **highly relevant** to the proposed research in depth and breadth<br>• has **all** the required skills and expertise to achieve the expected outcomes<br>• is **well networked** with domestic and/or international alliances to exchange knowledge and skills.
**5**<br>***Very Good***	The **research proposal**:<br>• is **relevant** to the call<br>• is **likely to be feasible**<br>• has objectives that are **clear** and **logical** that advances knowledge<br>• raises a **few minor concerns** with respect to the study design<br>• has **clear** strategies for performance measures/milestones and how grant funds and other resources will be shared, deployed, and redeployed<br>• will provide **some high quality** evidence that addresses the expected outcomes of the TCR<br>• may **not be highly competitive** with comparable research proposals internationally<br>• may be translated into, or inform changes in, policy or health practice<br>• may result in **several** influential publications	The **applicant team:**<br>• has a track record that is **very relevant** to the proposed research in depth and breadth<br>• has **most of** the required skills and expertise to achieve the expected outcomes<br>• is **networked** with domestic and/or international alliances to exchange knowledge and skills.
**4**<br>***Good***	The **research proposal**:<br>• is somewhat **relevant** to the call<br>• raises **some concerns** regarding feasibility<br>• has objectives that are **clear** that advance knowledge<br>• raises **some concerns** regarding the study design<br>• has **some** strategies for performance measures/milestones and how grant funds and other resources will be shared, deployed, and redeployed<br>• will provide **some evidence** that addresses	The **applicant team:**<br>• has a track record that is **consistent** with the proposed research in depth and breadth<br>• has **some** of the required skills and expertise to achieve the expected outcomes<br>• is **somewhat networked** with domestic and/or international alliances to exchange knowledge and skills.

	the expected outcomes of the TCR<br>• is **not likely to be competitive** with similar research proposals internationally<br>• may be translated into, or inform changes in, policy or health practice<br>• may result in **some** strong or influential publications	
**3**<br>***Marginal***	The **research proposal**:<br>• is **not particularly relevant** to the call<br>• raises **several concerns** regarding feasibility<br>• is **somewhat unclear** in its objectives and potential to advance knowledge<br>• raises **several concerns** regarding the study design<br>• has **superficial** consideration of strategies for performance measures/milestones and how grant funds and other resources will be shared, deployed, and redeployed<br>• may provide **limited** evidence that addresses the expected outcomes of the TCR<br>• is **not competitive** nationally or internationally<br>• is **unlikely** to be translated into, or inform changes in, policy or health practice<br>• may result in some **modestly** influential publications	The **applicant team:**<br>• has a **limited** track record in the field of the proposed research<br>• has **minimal** skills and expertise required to achieve the expected outcomes<br>• is **marginally networked** with domestic/and or international alliances to exchange knowledge and skills.
**2**<br>***Unsatisfactory***	The **research proposal**:<br>• raises **several major concerns** regarding feasibility<br>• is **unclear** in its objectives and capacity to advance knowledge<br>• raises **several major concerns** regarding the study design<br>• has **no** shared budget, **some** evidence of shared resources, **no** consideration of how grant funds and other resources will be shared, deployed, and redeployed<br>• has **little relevance** to the call<br>• is **not competitive** nationally or internationally<br>• is **unlikely** to provide evidence that addresses the expected outcomes of the TCR<br>• is **very unlikely** to be translated into, or inform changes in, policy or health practice<br>• is **unlikely** to result in influential publications	The **applicant team:**<br>• has a track record which **does not relate well** to the proposed research<br>• is **deficient in many** of the required skills and expertise to achieve the expected outcomes<br>• is **poorly networked** with domestic and/or international alliances to exchange knowledge and skills.
**1**<br>***Poor***	The **research proposal**:<br>• contains a research plan that **does not seem to be feasible** and is **unlikely** to be successfully completed	The **applicant team:**<br>• does **not have a relevant** track record in the field of the proposed research<br>• is **deficient in most** of the required skills

	• is **very unclear** in its objectives, and unlikely to advance knowledge<br>• contains a study design that is **not adequate**<br>• has **no** shared budget, **no** evidence of shared resources, **no** consideration of how grant funds and other resources will be shared, deployed, and redeployed<br>• is **not relevant** to the call<br>• is **not competitive** nationally or internationally<br>• is **very unlikely** to provide evidence that addresses the expected outcomes of the TCR<br>• has **no potential** to be translated into changes in or inform policy or health practice<br>• is **very unlikely** to result in influential publications	and expertise to achieve the expected outcomes<br>• is **not networked** with international alliances.

Consumer and community representatives (20% of overall assessment score[8])

Assessment criteria

Consumer and community involvement activities - evidence that consumers and/or the community were consulted/involved in the design of the research proposal, including detail of what ongoing community/consumer involvement will occur during the research and methods for communication of results.

Support for consumer and community involvement - the research team's demonstrated experience in working with consumers and communities and the provision of governance arrangements to support involvement (i.e. consumer input to advisory committees, planned activities or specific roles for community members), as well as adequate time and resourcing to involve and support the involvement of consumer and community representatives in the research.

Relevance and research impact for consumers and community - the proposed research addresses the objectives of the call in a way that is relevant to the needs of consumers and the community, and gives appropriate consideration to the needs of people from different and diverse cultural backgrounds or who have a higher disease burden or poorer health outcomes. The outcomes of research will make a difference by improving health services or health policy that will lead to improved health outcomes for individuals.

Category	***Consumer and community involvement activities***	***Support for consumer and community involvement***	***Relevance and research impact for consumers and community***
**7**<br>***Outstanding***	The research proposal details **outstanding** consumer and community involvement in:<br>• its design	The team has an **outstanding** track record in working with consumers and communities.<br>The research proposal provides **near flawless** arrangements	The research proposal:<br>• is **highly relevant** to the call objectives

[8] The three CCR criteria are equally weighted across the overall 20% contribution.

	• the proposed research activities<br>• communicating the outcomes to the participants.	and structures for coordinating consumer and community involvement in the research team, based on the:<br>• allocated budget<br>• governance arrangements<br>• administrative support<br>• training available.	• is **outstanding** in demonstrating how the research and potential outcomes meet the diverse needs of consumers and the community with consideration to culture, ethnicity, language and health status/needs<br><br>• will definitely lead to **major** and **effective** health gains across a broad range of groups<br><br>• has potential outcomes that will have a **very high impact** on improving health outcomes through improved health services delivery or policy.
**6**<br>***Excellent***	The research proposal details **excellent** consumer and community involvement in:<br><br>• its design<br>• the proposed research activities<br>• communicating the outcomes to the participants.	The team has an **excellent** track record in working with consumers and communities.<br><br>The research proposal provides **excellent** arrangements and structures for coordinating consumer and community involvement in the research team, based on the:<br>• allocated budget<br>• governance arrangements<br>• administrative support<br>• training available.	The research proposal:<br><br>• is **very relevant** to the call objectives<br><br>• is **excellent** in demonstrating how the research and potential outcomes meet the diverse needs of consumers and the community with consideration to culture, ethnicity, language and health status/needs<br><br>• will lead to **considerable** and **effective** health gains across a broad range of groups<br><br>• has potential outcomes that will have a **high impact** on improving health outcomes through improved health services delivery or policy.
**5**<br>***Very Good***	The research proposal details **very good** consumer and community involvement in:<br><br>• its design<br>• the proposed research activities<br>• communicating the outcomes to the participants.	The team has a **very good** track record in working with consumers and communities.<br><br>The research proposal provides **very good** arrangements and structures for coordinating consumer and community involvement in the research team, based on the:<br>• allocated budget<br>• governance arrangements<br>• administrative support<br>• training available.	The research proposal:<br><br>• is **relevant** to the call objectives<br><br>• is **very good in demonstrating** how the research and potential outcomes meet the diverse needs of consumers and the community with consideration to culture, ethnicity, language and health status/needs<br><br>• will lead to **effective** health gains across a broad range of groups<br><br>• has potential outcomes that **will have an impact** on improving health outcomes through improved health services delivery or policy.

**4** ***Good***	The research proposal details a **good** level of consumer and community involvement in:<br>• its design<br>• the proposed research activities<br>• communicating the outcomes to the participants.	The team has a **good** track record in working with consumers and communities.<br><br>The research proposal provides **good** arrangements and structures for coordinating consumer and community involvement in the research team, based on the:<br>• allocated budget<br>• governance arrangements<br>• administrative support<br>• training available.	The research proposal:<br><br>• is **somewhat relevant** to the call objectives<br><br>• is **good in demonstrating** how the research and potential outcomes meet the diverse needs of consumers and the community with consideration to culture, ethnicity, language and health status/needs<br><br>• **may** lead to effective health gains across a broad range of groups<br><br>• has potential outcomes that **may have an impact** on improving health outcomes through improved health services delivery or policy.
**3** ***Marginal***	The research proposal details **some** level of consumer and community involvement in:<br>• its design<br>• the proposed research activities<br>• communicating the outcomes to the participants.	The team has a **limited** track record in working with consumers and communities.<br><br>The research proposal provides **minimal** arrangements and structures for coordinating consumer and community involvement in the research team, based on the:<br>• allocated budget<br>• governance arrangements<br>• administrative support<br>• training available.	The research proposal:<br><br>• is **not particularly relevant** to the call objectives<br><br>• is **limited** in demonstrating how the research and potential outcomes meet the diverse needs of consumers and the community with consideration to culture, ethnicity, language and health status/needs<br><br>• may lead to **limited** health gains across a broad range of groups<br><br>• has potential outcomes that **may have a moderate impact** on improving health outcomes through improved health services delivery or policy.
**2** ***Unsatisfactory***	The research proposal details **limited** consumer and community involvement in:<br>• its design<br>• the proposed research activities<br>• communicating the outcomes to the participants.	The team has an **unsatisfactory** track record in working with consumers and communities.<br><br>The research proposal provides **inadequate** arrangements and structures for coordinating consumer and community involvement in the research team, based on the:<br>• allocated budget<br>• governance arrangements<br>• administrative support<br>• training available.	The research proposal:<br><br>• is **not adequately relevant** to the call objectives<br><br>• is **deficient** in demonstrating how the research and potential outcomes meet the diverse needs of consumers and the community with consideration to culture, ethnicity language and health status/needs<br><br>• is **unlikely** to lead to any health gains across a broad range of groups<br><br>• has potential outcomes that are **unlikely to have an impact** on improving health outcomes through improved health services delivery or policy.

**1**<br>***Poor***	The research proposal details **no** consumer and community involvement in:<br><br>• its design<br>• the proposed research<br>• communicating the outcomes to the participants.	The team has **no demonstrated** track record in working with consumers and communities.<br><br>The research proposal provides **no** arrangements or structures for coordinating consumer and community involvement in the research team, based on the:<br>• allocated budget<br>• governance arrangements<br>• administrative support<br>• training available.	The research proposal:<br><br>• is **not relevant** to the call objectives<br><br>• **does not** demonstrate how the research and potential outcomes meet the diverse needs of consumers and the community with consideration to culture, ethnicity, language and health status/needs<br><br>• **will not** lead to any health gains across a broad range of groups<br><br>• has potential outcomes that **will not have an impact** on improving health outcomes through improved health services delivery or policy.

Appendix B. TCR: Cultural, ethnic and linguistic diversity in dementia research Guide to Applicants

1. Preparing an application

The following sections provide additional advice about parts of the application that are specific to TCR: Cultural, ethnic and linguistic diversity in dementia research 2022 grant opportunity.

- Refer to the *Sapphire Learning and Training Resources* for general instructions on how to apply for a grant in Sapphire.
- TCR: Cultural, ethnic and linguistic diversity in dementia research 2022 grant opportunity scheme-specific policy and instructions for applying in Sapphire (grey boxes) are provided in this Appendix.
- For further assistance during the application process, refer to Section 7 - How to apply in the grant opportunity guidelines.

2. Application Requirements

A complete application is comprised of:

- Completed mandatory sections of 'My Profile' (Appendix B, Section 4) and 'My Profile', Requirements for TCR: Cultural, ethnic and linguistic diversity in dementia research (Appendix B, section 5).
- Completed application form (Appendix B, Section 6)
- Grant Proposal as an attachment (Appendix B, Section 6.7)

Applications must comply with all requirements as set out in the grant opportunity guidelines. Failure to adhere to any of these requirements may result in non-acceptance or exclusion of your application (refer to Section 4 Eligibility criteria of the guidelines).

2.1 Use of gender-neutral language

The vision of the NHMRC Gender Equity Strategy 2022-2025https://www.nhmrc.gov.au/about-us/publications/nhmrcs-gender-equality-strategy-2018-2021 is a gender diverse and inclusive health and medical research workforce to take advantage of the full range of talent needed to build a healthy Australia. Using gender-neutral language is one strategy to support this goal, serving to de-emphasise gender in the assessment of grant applications and reduce the potential impact of unconscious bias.

NHMRC strongly encourages the use of gender-neutral language in applications. This means that, wherever possible and appropriate, applicants should avoid the use of words that reveal their gender or the gender of team members. These words include (but are not limited to) her, him, she, he, Mr, Ms, Mrs and Miss, as well as first names and terms such as 'maternity leave'.

It is recognised that there will be instances where reference to the gender of applicants or team members is unavoidable or desirable.

However, wherever possible and relevant, applicants should:

- use the first person, *i.e. I/me/my*, rather than referring to themselves in the third person, or if third person is preferred, then use CI last-name or CIA
- use CIB, CIC, etc. or plural pronouns, *i.e. their/they,* when referring to others, rather than he/she or her/his
- use the format 'CI last-name', *e.g. CI Jones,* rather than using first names when referring to individuals

- use gender-neutral nouns, *e.g. researcher, staff,* etc., including when completing career disruption information, if relevant, *e.g. parental leave,* rather than maternity/paternity leave,
- review the application for instances of 'masculine form by default' before submission and remove them, e.g. 'every team member will manage his data according to this protocol' can instead use the plural pronoun *their*.

Note the aim is to de-emphasise **applicant** gender. Where gender is important for the research being proposed, it should be included in the application.

2.2 Minimum Data Requirements

Minimum data must be entered in Sapphire by the specified due date. Applicants must complete the required fields with correct information and are discouraged from making changes to this information after the minimum data due date. NHMRC uses this information to identify peer reviewers who are best suited to assess the application.

Minimum data are indicated in Sapphire by a blue flag (⚑) and are comprised of:

- Administering Institution
- Aboriginal and/or Torres Strait Islander Health Research Focus (yes/no)
- Project synopsis
- Privacy agreement
- Research Classification:
    - Broad research area
    - Field(s) of research
    - Peer Review Areas
    - Research keywords
- All Chief Investigators listed on the application (complete CI Role and Name) Note – CIB-J can be changed after minimum date close, up until round close.

Minimum data must be entered into Sapphire by 02 November 2022. Applicants are to refer to Section 7.3 Minimum data of the guidelines for further information.

<u>Failure to meet this deadline will result in the application not proceeding.</u>

Research Administration Officers (RAOs) are not required to certify applications for the purpose of minimum data. Applications only require certification once complete and ready for submission to NHMRC.

3. Key Changes

Applicants: please note the following changes for the TCR: Cultural, ethnic and linguistic diversity in dementia research 2022 grant opportunity application form:

- Applications submitted to this TCR will be assessed by a peer review panel comprised of two groups of peer reviewers:
    - Health and medical researchers
    - Consumer and community representatives (CCRs).

Previous TCRs have included CCRs as members on peer review panels, providing scoring members of the panel with qualitative feedback on how well applicant teams have involved consumer and community perspectives in their research proposals. For this TCR, CCRs will provide a score that contributes to 20% of the overall assessment score. There are dedicated assessment criteria and category descriptors for each peer reviewer group. For more detail see Section 6 and <u>Appendix A</u>.

- Chief Investigators (CIA-CIJ) are required to nominate up to 10 of their best publications from the past 10 years, taking into account career disruption, within the Sapphire

application form. This information will be used by the health and medical research peer reviewers to assess track record.

4. 'My Profile' Requirements

Within your profile in Sapphire, there is mandatory information that must be provided and/or updated before an application is submitted (refer to Section 7 How to apply of the guidelines). This information includes personal details, academic/research interests, and peer review information.

Mandatory Profile information is indicated by a red asterisk in Sapphire (*). This requirement applies to all Chief Investigators (CIs) named on the application. Please verify that each of the CIs has completed and/or updated their profile before an application is certified, noting that an error message will appear when a CI is added as a team member if they have not completed all mandatory fields in their profile. Existing NHMRC grant holders cannot commence or be named on an application until all mandatory 'My Profile' fields are complete.

4.1 About My Profile

Provide your primary institution name under Primary Institution. If this is an Administering Institution, the RAO will have access to view your profile; you may also allow the RAO to edit your profile.

Note: to update your Primary Institution name in Sapphire, go to 'Account Settings', 'Personal details' and click on 'Primary Institution'.

4.2 Personal information

Provide your most current details in this section. It is important that your title, names, phone and email details are up to date as these are the details on which NHMRC relies to contact you.

Aboriginal and Torres Strait Islander health research and researchers

Applicants identifying as being of Aboriginal and/or Torres Strait Islander descent are asked to indicate this in their Sapphire profile.

Identification of Aboriginal and/or Torres Strait Islander descent follows the advice provided on the AIATSIS website (https://aiatsis.gov.au/family-history/you-start/proof-aboriginality). This states that government agencies and communities usually accept three 'working criteria' as confirmation of Aboriginal or Torres Strait Islander heritage, namely:

- being of Aboriginal or Torres Strait Islander descent
- identifying as an Aboriginal or Torres Strait Islander person, and
- being accepted as such by the community in which you live, or formerly lived.

Administering Institutions must retain evidence, consistent with AIATSIS guidance, of a research team member's identification as an Aboriginal and/or Torres Strait Islander person and must provide this evidence to NHMRC, if requested.

4.3 Academic Information

Indicate whether you have a Doctor of Philosophy (PhD) and, if applicable, the pass date (year) of your thesis (not the date of conferral).

4.4 Peer Review Information

Select a Broad Research Area and 5-10 Research Keywords most applicable to your main area of research. You can also provide further detail about your research interests or areas of expertise. This could include, but is not limited to, your research methodologies, areas of student supervision and areas in which you have published.

Select up to three (3) Peer Review Areas (PRAs) that best describe your research interests, 1 being the most relevant and 3 being the least relevant.

You can add as many Fields of Research as required. Indicate when you started your research in that field, the classification of the research (e.g. primary), and whether the research is current or terminated. Individuals are encouraged to list all Fields of Research. Only current Fields of Research will be displayed.

Note: An opportunity is provided in the application to select research areas, fields of research and keywords that best describe your research proposal, as opposed to your personal research interests. The above information about your personal research interests will not determine the peer reviewers selected for your application.

4.5 Unavailability Calendar

Peer review is an integral part of NHMRC funding schemes. NHMRC grant recipients have obligations to contribute to the assessment of applications (as outlined in the *NHMRC Funding Agreement*). If you are not available to act as a peer reviewer, please provide a statement detailing your reasons and the period for which you are unavailable. To maintain the list of available peer reviewers within Sapphire, NHMRC requests that all applicants update their availability routinely. This will avoid unnecessary contact if you are unavailable.

4.6 Contributions to NHMRC

Please indicate in which role you have contributed to NHMRC, if you have previously participated in an advisory, peer review, guideline development or other NHMRC activity requiring expert stakeholder input via formal appointment.

Click '+' to start a new entry to specify the below:

- Select a 'Contribution Role', from the drop-down menu
- Indicate the year in which you held the Contribution Role.
- Indicate the number of times you acted in that role in each year.

You will need to create a new entry for each type of contribution in a particular year.

5. 'My Profile' Requirements specific to TCR: Cultural, ethnic and linguistic diversity in dementia research 2022 grant opportunity

The following sections provide advice about parts of the application that are specific to TCR: Cultural, ethnic and linguistic diversity in dementia research 2022 grant opportunity. For the purposes of this grant opportunity, you are only required to complete the sections outlined below. If you enter more information than is required, only the required information will be imported into your application.

It is important that relevant 'My Profile' information (for all CIs) is up to date at the time of application submission, as it is used to contact applicants, imported into the application and used by peer reviewers. It may also be used for analyses of NHMRC's funding profile and to

capture grant outcomes. ‘My Profile’ information can be updated at any time. However, any changes made to ‘My Profile’ (for any CI) after Chief Investigator A (CIA) certification will not appear in the submitted application.

Instructions for entering ‘My Profile’ information in Sapphire are provided in the *Sapphire Learning and Training Resources*.

Note: You are required to list research outputs in relevant subsections of your profile. You are encouraged to link the entered research output to NHMRC Grant IDs, where applicable.

5.1 Career Disruptions (within the last 5 years)

NHMRC is committed to ensuring that every applicant is treated fairly, and this means that it recognises some applicants will have had career disruptions that need to be considered when evaluating their track record and eligibility. If applicable, applicants should use this opportunity to declare any career disruptions that may be relevant to their career history.

The period of career disruption may be used to determine an applicant’s eligibility for a grant opportunity or to allow additional track record information to be considered during assessment.

Career Disruption

A Career Disruption is defined as a prolonged interruption to an applicant’s capacity to work due to pregnancy, major illness/injury and/or carer responsibilities. For guidance on what constitutes a Career Disruption and how it is considered, refer to the NHMRC Relative to Opportunity Policy.

Career Disruption claims will not be considered for applications that fail to comply with the following requirements:

The last 5 years of Career Disruptions will be included for each CI and provided to peer reviewers for assessment.

Disruption Type
To enter a Career Disruption, click ‘+’. Select a ‘Disruption type’ from the drop-down menu.

Impact
Provide a brief explanation of the impact the Career Disruption(s) has had on your research, research achievements and associated productivity relative to your career stage. Include the percentage (%) full-time equivalent (FTE) of the Career Disruption. Do not describe the nature of the Career Disruption in this field.

Note that the information in this field will be provided to peer reviewers.

Additional Research Outputs
Provide details of publications that you would like to claim in relation to this Career Disruption.

Note - It is permissible to include these publications in your top 10 publications for the past 10 years (see Section 6.5 – Research Team) if they were published in the period being claimed. For example, if an applicant is claiming 2 years’ career disruption, they are permitted to include publications from the past 12 years. Please note - A maximum of 15 years applies (10 + 5 years career disruption).

Dates
Nominate the periods when you have had a disruption (approximate dates). Entries will be listed in reverse chronological order.

5.2 Relative to Opportunity (within the last 5 years)

If applicable, the applicant can use this section to provide details of any Relative to Opportunity considerations and the effect they have had on their research and research achievements,

including (but not limited to) interruptions due to calamities e.g. bushfires and the COVID-19 pandemic.

The last 5 years of Relative to Opportunity information will be included for each CI and provided to peer reviewers for assessment.

Circumstances
Provide a brief explanation of the type of Relative to Opportunity circumstance.

Impact
Provide a brief explanation of the impact this has had on your research, research achievements and associated productivity relative to your career stage.

Date
Nominate the periods when you have had a Relative to Opportunity circumstance (approximate dates). Entries will be listed in reverse chronological order.

5.3 Community Engagements (within the last five years)

Click '+' to start a new entry for any community engagement that you have been involved in. You may wish to consult the Statement on Consumer and Community Involvement in Health and Medical Research which has been developed to recognise the contribution that consumers can make to health and medical research and their right to do so. The Statement is available on the NHMRC website.

5.4 Translation into Policy/Practice (within the last five years)

Click '+' to start a new entry for any activities which have resulted in research translation.

Provide details of any of your research that has resulted in changes to organisational or government policy, health care systems, delivery of health care, treatments or interventions in diseases and conditions, and professional development for health practitioners. In addition to the impact on health policy, you can also include, if applicable, the wider impact that your research has had on policy and practices that affect society and the economy. Entries will always be listed in reverse chronological order.

Name

Provide a relevant short name for the impact of your research on policy or practice.

Research

Provide a brief description of your research that led to this impact on policy or practice and the resulting outcomes.

Changes

Provide a brief description of the outcomes resulting from your research.

Description of Impact

Provide details of the organisation, government department etc. that benefited from the research.

Sector Affected

Name the sector(s) affected by your research and its resultant translation into policy/practice.

Other details

Select from the drop-down list your role, the impact on either policy or practice and the type of impact. Indicate the year the change was translated/implemented and provide details of the resulting changes. Indicate if the research was funded by NHMRC, another Australian agency or an international source.

If the research was funded by NHMRC, please select the NHMRC grant underpinning the research in the ‘Related Grants’ field.

Note: For schemes that limit the information presented to peer reviewers (e.g. “last five years”), the time period will be based on the date of translation (year of change), NOT the date of the original research.

You will need to create a new page for each impact.

6. Application Form Requirements

The following sections of the application form are specific to TCR: Cultural, ethnic and linguistic diversity in dementia research grant opportunity and must be completed as part of your application. Step-by-step instructions for entering application details in Sapphire are provided in the *Sapphire Learning and Training Resources*.

6.1 Creating an application

Click ‘’+ New Application’ to create an application.

Grant Opportunity

Select the grant round you wish to apply for, e.g., TCR: Cultural, ethnic and linguistic diversity in dementia research 2022. The application title will be used to identify the application at all times during the assessment process and needs to accurately describe the nature of the research proposal.

6.2 Application details

All fields on this page marked with a flag (🏴) must be completed to meet minimum data requirements.

Application Identification Number (APP ID)

Each application will have its own unique Application Identification Number (Application ID), which is automatically generated by Sapphire and pre-filled in the application. Use this Application ID number (e.g. 2345678) to identify your application when referring to it in any correspondence.

Administering Institution

Select your Administering Institution by entering three characters to start searching. There can be only one Administering Institution for each application. You must ensure that the institution you choose as your Administering Institution is the correct institution for your application. If in doubt, contact the RAO at your proposed Administering Institution.

Grant Duration

This section may contain pre-filled information that cannot be edited. If not, select the requested duration of your grant (in years) with reference to any limits specified in the grant opportunity guidelines.

Aboriginal / Torres Strait Islander Health Research

This question enables you to identify research that specifically investigates Aboriginal and Torres Strait Islander health issues. It is also designed to enable NHMRC to identify those research proposals that will require assessment of the proposed research against the *Indigenous Research Excellence Criteria*.

Only select ‘Yes’ if you can demonstrate that at least 20% of your research effort and /or capacity building relates to Aboriginal and Torres Strait Islander health.

If you have answered ‘Yes’ to this question, you will be required to provide details of how your application addresses the *Indigenous Research Excellence Criteria* in the application form. Your application may be assessed against the *Indigenous Research Excellence Criteria*, using information you provide in the following text boxes: ‘Community Engagement’, ‘Benefit’, ‘Sustainability and Transferability’ and ‘Build Capacity’.

Project Synopsis

The synopsis should accurately, and briefly, summarise the research proposal. This information may be used to assign applications to panels and peer reviewers. It may also be considered in the peer review process.

Plain English Summary

Describe the overall aims of the research and expected outcomes in simple terms that could be understood by the general public. Avoid the use of highly technical terms. This information may be used in grant announcements, media releases and other public documents, and by funding partners (where applicable) to determine whether the research proposal meets their priorities for funding.

Privacy Agreement

NHMRC, as an agency subject to the Privacy Act 1988 (Cth), is required to notify you about our collection, use and disclosure of your personal information. We do so by referring you to the NHMRC Privacy Policy (NHMRC Privacy Policy). Please ensure that you have carefully read and understood the Privacy Policy before completing the application. If you require further clarification, please contact the NHMRC Privacy Contact Officer via email (NHMRC.Privacy@nhmrc.gov.au) or letter (NHMRC, GPO Box 1421, Canberra ACT 2601).

Have you read and understood the NHMRC Privacy Policy?

Select ‘Yes’ or ‘No’.

In addition, and in accordance with Australian Privacy Principle 8 in the Privacy Act 1988 (Cth), we seek your consent to send your personal information (consisting of an "Assessor Snapshot Report") overseas, for the purposes of peer-review of this application if required. NHMRC uses the expertise of some peer assessors who reside overseas. While we make every effort to protect your personal information, assessors outside Australia are bound by their own country's laws and consequently we cannot provide assurance that your information will be handled in accordance with the same standards as required by the Privacy Act 1988, or that you would have similar remedies if your personal information is released in breach of local privacy laws.

Select ‘Yes’ or ‘No’.

Partner organisation consent

Do you give consent to provide your application and assessment results to other partner organisations?

Select ‘Yes’ or ‘No’.

If you wish to be considered for funding by a partner organisation, select ‘Yes’. By selecting ‘Yes’ you are consenting to NHMRC providing your application and/or assessment information

to potential funding partners if your application fits the funding partner's research funding objectives. For a list of funding partners, please refer to this grant opportunity's information on GrantConnect.

6.3 Participating Institutions

In some cases, the institution that will administer your application may differ from the institution in which you will actually conduct the proposed research or your proposed research may be conducted at a collaborating institution in addition to your administering institution. For example, many universities administer research that will be conducted in an affiliated teaching hospital. Information on 'Participating Institutions' is required by NHMRC to enable peer reviewers to identify potential institutional conflicts with your application and for grant administration purposes

Research Institution

List the Participating Institution and department where the proposed research will be conducted.

To add more than one Participating Institution, press '+' and complete the required information. If the participating institution does not appear in the list, please email the institution name to the Research Help Centre (help@nhmrc.gov.au).

Research Effort (%)

If the research will be conducted at more than one institution, enter the Research Effort percentage (%) allocated to each participating institution and department. The percentages (%) entered must total 100%.

NOTE: If some or all of the proposed research will be carried out at your Administering Institution, create an entry with the Administering Institution and choose a percentage up to 100%. At least one institution must be listed.

6.4 Research Classification

The details entered in this section will be used in the peer review process to assist with the selection of appropriate peer reviewers for your application. It may also be used for analyses of NHMRC's Funding Profile.

All fields on this page marked with a flag (⚑) must be completed to meet minimum data requirements. You must make the selections that best describe your research proposal against each of the following fields:

- Broad Research Area
- Field(s) of Research
- Peer Review Areas
- Research Keywords
- Burden of Disease

Click '+' to add rows for each Field of Research that best describes the area of research of the application. You can select up to three Fields of Research.

Select a Burden of Disease that best describes the area of research of the application. You can select up to three Burden of Disease types. Click '+' to add rows for each additional

Burden of Disease. You must allocate a percentage (%) of time against each. The percentage (%) total must not exceed 100%.

6.5 Research Team

You can include a maximum of 10 Chief Investigators (CIs) and 10 Associate Investigators (AIs) in your research team. For further information of the eligibility requirements for CIs and AIs, please refer to Section 4 Eligibility criteria of the guidelines.

All fields on this page marked with a flag (🏴) must be completed to meet minimum data requirements. List all members of research team, including CIs and AIs. Complete a separate entry for each member of the team by clicking '+' to add rows.

All CIs/AIs must have a Sapphire account in order to be listed as part of the Research Team. CIs/AIs who cannot be located using the search function will need to complete registration. Submission of a registration form and activation of a Sapphire account must occur at least 72 hours before application close, noting that account activation processes cannot be guaranteed within this time.

Note: Click 'Invite to Register & Manage Access' to invite a Chief/Associate Investigator to complete Sapphire Registration and/or share your application with view or edit access. Enter the email address, select the corresponding access option from the drop-down menu and click 'Save and send'. Users will receive an email invitation to be assigned to the application with appropriate access rights. Click '+ Add another user' to invite any additional Investigators.

Please ensure that you have the correct email addresses for your colleagues before commencing your application.

Chief Investigators (CIA-CIJ)

The 'Role' and corresponding 'Name' fields for Chief Investigator A must be completed to meet minimum data requirements. If you are naming yourself for a CI/AI role, 'Invitation Response' status will automatically change to Accepted.

Indicate whether the Chief Investigator A will be based in Australia for the duration of the grant and outline their background and expertise relevant to the grant proposal.

Click '+'to add rows for additional CIs. Click the 'Role' drop-down to select a role for the CI.

To add a CI to your research team, enter their email address. If they are a registered user, you can click 'Send invite'. If the user is not found, please invite them to register from the button 'Invite to Register & Manage Access' at the top. Outline the background and expertise relevant to the grant proposal for each additional Chief Investigator.

If you add a CI to your research team, an email will automatically be generated to the team member for their agreement to be named on the application. The invitation response status next to their name will indicate progress. Invitations must be accepted by CIs in order for applications to be submitted.

Note: Emails to added CIs will be sent after a short delay. Invitation status will not update to *Sent* until you have logged out of the application for 15 minutes.

Associate Investigators (AIs)

Click '+' to Add Rows for AIs. To add an AI to your research team, enter their email address. If they are a registered user, you can click 'Send invite'. If the user is not found, please invite them to register from the button 'Invite to Register & Manage Access' at the top.

'Position' is optional. 'Relevant background and expertise' is optional.

If you add an AI to your research team, an email will automatically be generated to the team member for their agreement to be named on the application. The invitation response status next to their name will indicate progress. Invitations must be accepted by AIs in order for applications to be submitted.

Note: Emails to added AIs will be sent after a short delay. Invitation status will not update to *Sent* until you have logged out of the application for 15 minutes.

Publications

Applicants are required to nominate up to 10 of their best publications from the past 10 years (taking into account any career disruptions). You are to provide separate explanations for each citation (publication) entry. Each explanation should explain why the publication has been selected, including its quality and contribution to science, and your contribution to each.

You may include field weighted metrics and citation metrics within the explanation field for the 10 best publications from the last 10 years. Where possible, references to publications within the provided entry fields should be provided as a complete citation. Where this is not possible, include sufficient citation information to locate the publication such as digital object identifier, journal name, publication title, year and authors.

Publications outside the last 10 years (taking into account any career disruptions) and other research outputs such as patents can be referred to in the Research Impact section if relevant.

The assessment of publications will be against the category descriptors at Table 2 of Appendix A.

Top 10 in 10

Provide the details of (up to) 10 of your best publications in the last 10 years.

Each publication should be provided separately, i.e. one publication per free-text field, with an explanation as to why the publication was nominated:

- Publication 1 - 10 (max. 200 characters for citation)
- Explanation (max. 2000 characters).

6.6 Ethics

If you answer "Yes" to any of the questions, you will need to obtain ethics approvals and supply evidence of these to your research office in the event your application is funded. For further information, see *Ethics and Integrity* on the NHMRC website.

6.7 Grant Proposal

Applicants must not include in any part of their application:

- Links to external websites, apart from references to journal articles, guidelines, government reports, datasets and other outputs that are only available online; where links are included, provide the URL in full (e.g. the NHMRC website https://www.nhmrc.gov.au). Applicants are asked not to use URL shorteners as this may create a security risk.
- Publication metrics such as Journal Impact Factors, consistent with the recommendations in the San Francisco Declaration on Research Assessment.

The grant proposal must be written in English and submitted in a Portable Document Format (PDF) file, using NHMRC's Grant Proposal template, which will be available on GrantConnect. Applicants must use this template. The grant proposal must be uploaded into Sapphire.

Grant Proposal (Upload)

To upload your Grant Proposal PDF, select the 'Upload New' button followed by the 'Choose File' button. Select the PDF file you wish to upload and then click 'Start upload' to upload your Grant Proposal. Click 'Save' to ensure the application is submitted correctly.

To ensure that the document is displaying properly, applicants need to open a copy of the uploaded document by selecting the open icon to the right of the document name after the document has been saved in Sapphire.

Naming and formatting requirements for the grant proposal, to ensure fairness and consistency across applicants, are listed in Table 1. Applications that fail to comply with these requirements may be excluded from consideration.

Details to be addressed in the grant proposal and associated page limits are set out in Table 2. Applicants should note that peer reviewers will, as part of their assessment, consider the reproducibility and applicability of the proposed research and research design. Within the experimental design of the proposal, applicants need to include sufficient information to demonstrate that robust and unbiased results will be produced.

Table 1: Formatting Requirements

Component	Component Requirements
File format	The grant proposal must be saved and uploaded as a PDF file
File size	The PDF file MUST NOT exceed 2 MB in size
File name	The PDF file must be named using the following:<br>Applicant's Surname_Document Type/Name.pdf<br>E.g.: Smith_Grant Proposal.pdf
Page size	A4
Header	Application ID and Applicant surname must be included in the header
Footer	Page number must be included in the footer
Font	NHMRC recommends a minimum of 12-point Times New Roman font. Applicants must ensure the font is readable.
Margins	Pages must have 2 cm top, bottom, left and right margins.
Line spacing	Single
Language	English

Table 2: Grant Proposal Components

Component	Page Limit
Research Proposal	9 pages
References	2 pages
Community and Consumer Involvement Summary	3 pages

Team quality and capability relevant to the proposal	1 page
CIs Track Record	2 pages per CI

A brief description of each component is provided below.

Research Proposal – 9 pages

This section should address the following assessment criteria:

- Scientific quality and relevance to the objectives and expected outcomes of the proposed research (50%)
- Team capacity and record of achievement of the team in areas/disciplines relevant to this TCR – relative to opportunity (30%).

The research proposal must address the essential components of your research and can include the following properties depending on the type of research:

**Component**	**Properties**
Aims	Describe the specific aims of the project, including a clear statement of hypotheses to be tested.
Background	Provide a rationale for the project.
Research Plan – methods and techniques to be used	Outline the research plan in detail, including the following where appropriate:<br>- detailed description of the experimental design<br>- techniques to be used<br>- details and justification of controls<br>- details for appropriate blinding<br>- strategies for randomisation and/or stratification<br>- justification of sample-size, including power calculation<br>- justification of statistical methods<br>- strategies to ensure that the experimental results will be robust, unbiased and reproducible<br>- details to achieve balance of male and female study participants, and male and female cell and animal models, including justification where it is not warranted<br>- any ethical considerations<br>- community involvement and/or plans to transfer knowledge to stakeholders or into practice

Component	Properties
	- strengths and weaknesses of the study design and approach
Timeline	Provide a detailed timeline for the expected outcomes of the Research Proposal along with justification for the duration requested.
Identified Risks	Describe the scientific and/or technical risks associated with the research plan and how these will be managed. This could include risks and mitigations relating to the known or anticipated impact of COVID-19 on the research plan.<br>Include details, if applicable, of how Associate Investigators (AIs) help to mitigate or control any risk.
Outcomes and Significance	Describe the importance of the problem to be researched, the planned outcome of the research plan, and the potential significance of the research.

References cited in this document are to be listed in the separate References section.

References – 2 pages

References for the Research Proposal must:

- not exceed 2 pages
- provide a list of all references cited in the application in an appropriate standard journal format (NHMRC prefers the Author-date (also known as the Harvard System), Documentary-note and the Vancouver Systems)
- list authors in the order in which they appear in PubMed
- only include references to cited work
- must be written in English.

Consumer and Community Involvement Summary – 2-3 pages

This section should address the following assessment criteria:

- Consumer and community involvement activities
- Support for consumer and community involvement
- Relevance and research impact for consumers and community.

This section provides a summary of the research proposal and specific details relevant to the consumer and community representatives who will assess the application against the above assessment criteria. Applicants must:

- provide a plain English summary of their research proposal detailing what the research aims to achieve and the significance in relation to the call
- outline:
    - consultation and involvement of consumers (patients, their families, carers, consumer representatives or organisations) and the wider community in designing and planning the proposed research

    - the relevance of the proposed research, its potential impact and how it meets the diverse needs (e.g. burden of disease, accessibility of health services, cultural background, socioeconomic status) of the target consumers and community
    - previous track record of working with consumers and communities to undertake research activities
    - any governance arrangements for consumers and/or community involvement in the research team or research activities
    - details of resourcing (financial, time, in-kind) planned to support the involvement of consumer and community representatives in the research project.
- ensure that all key terms and acronyms used are spelt out and, if appropriate, explained
- not exceed 3 pages.

Team Quality & Capability relevant to this application, relative to opportunity (does NOT include Associate Investigators) – 1 page
A summary of the research team's quality and capability must be contained in this section. Applicants should detail:

- the expertise and productivity of team members relevant to the proposed project
- their influence in this specific field of research
- how the team will work together to achieve the project aims
- how junior members are contributing to the overall track record of the team.

CI Track Record – 3 pages per CI
Reviewers will use this section to assess the track record quality of the research team.

Applicants are encouraged to use this section to identify aspects of their track record that are in addition to those that have been listed in the My Profile section (see Appendix B, Section 4 and 5) and the Top 10 publications in the last 10 years (see Appendix B, Section 6.5). This includes any relative to opportunity considerations you wish the assessors to take into consideration (please see Appendix B). The following areas should be considered (if relevant to the proposed research):

- Career summary – including qualifications, employment and appointment history
- Contribution to field of research – this may include the impact of previous research including translation of health research into implementable, tangible outcomes
- Patents – this information should include if the patent has been licensed, when they have been licensed, to whom they have been licensed and if that licence is current or not
- Collaborations
- Community engagement and participation, including previous engagement with patient and support groups
- Professional involvement – including committees, conference organisation, conference participation
- International standing
- Supervision and mentoring
- Peer review involvement (including NHMRC, other granting organisations, manuscripts, editorial responsibilities)
- Industry relevant expertise and output
- Other contributions to NHMRC
- Other information you think is vital to your application.

6.8 Third Party Research Facilities

Applicants often need to receive services from research facilities to undertake their research.

Such facilities include but are not limited to: biospecimens and associated data from biobanks or pathology services, non-human primate colonies, the Australian Twin Registry, Cell Bank Australia, and the Trans-Tasman Radio Oncology Group and other organisations that provide clinical trials services.

Applicants will need to consult with research facilities to ensure that the services they require can be provided and that the charges included in the budget are accurately reflected (refer to Direct Research Costs section). Letters from research facilities confirming their collaboration must be submitted with the application.

> Indicate whether you will be using services provided by a research facility to complete your research. If you select 'yes', then upload your letter from the research facility confirming their collaboration.
>
> To ensure that the document is displaying properly, applicants need to open a copy of the uploaded document by selecting the open icon to the right of the document's name after the document has been saved in Sapphire.

6.9 Direct Research Costs

Details on permitted uses of NHMRC funds and setting of budgets can be found in the *Direct Research Costs Guidelines* on the NHMRC website.

Salary

Salary contributions for research staff, including members of the research team, are provided as Personnel Support Packages (PSPs). The level of PSP requested in an application must match the roles and responsibilities of the position in the proposed research and the percentage of the PSP requested must reflect the required time commitment. Applicants must fully justify all requests for PSPs.

Further information about PSPs, including the levels, is available on the NHMRC website.

> This section only needs to be completed if you are seeking salary for a research role.
>
> **Position function**
>
> Describe the function of the research position for which a salary is requested.
>
> Note: A PSP is awarded based on a justified research function and it is not tied to an individual.
>
> **Salary level**
>
> Indicate the PSP level for the research position based on the level of the work to be undertaken and the % of a full PSP package to be paid for each year of funding (in whole numbers only). Applicants must apply for the exact proportion of a PSP that is required for the research being proposed.
>
> **Reason for salary**

Provide detailed justification for the salary that is being requested for the research position. The PSP level and the percentage of salary must both be well justified.

Note: When awarding a budget, peer reviewers will consider whether the PSPs requested are fully justified and reasonable given the time commitment indicated for this application.

Other Research Costs Provide details on:

- the name/description of the item
- the total value of the item requested for each year
- the justification for the particular item requested.

This information must be aligned with the proposed aims of the study, be detailed on a yearly basis and be fully justified (including, in the case of equipment costing less than $10,000, why the equipment cannot be provided by the institution).

Equipment Provide details on:

- the name/description of the item
- the total value of the item requested for each year
- the justification for the particular item requested.

Applicants can request funding to pay for equipment costing over $10,000 that is essential for the research. The total equipment requested cannot exceed $80,000. Individual items of equipment costing less than $10,000 must be requested within the 'Other Research Costs' category.

Applicants must clearly outline the total value of all items of equipment for each year, why the equipment is required for the proposed research and why the equipment cannot be provided by the institution.

For each item of equipment requested, a written quotation must be received and held with the RAO of the Administering Institution and be made available to NHMRC on request. The Administering Institution must be prepared to meet all service and repair costs for equipment funded.

Funds will not be provided for the purchase of computers except where they are an integral component of a piece of laboratory equipment or are of a nature essential for work in the research field, for example, a computer which is dedicated to data collection from a mass spectrometer or used for the manipulation of extensively large datasets (i.e. requiring special hardware).

Note: NHMRC funds the direct costs of research based on advice from peer review. Applicants should provide detailed justification of budgets requested. Inadequately justified budgets may be adjusted.

Funding cannot be used for infrastructure, particularly land, buildings and fixtures.

Entering Other Research Costs and Equipment Costs

You will need to create a separate entry for each cost. Click the plus (+) button to enter a cost.

For 'Item name', enter a brief name/description of the item.

Outline the cost of the item required for each year of the grant proposal. Only the relevant years need to be completed.

**Justification**<br>Provide a comprehensive justification for the cost.

7. Certifying your application

Once all 'My Profile' details, application form details and supporting documents have been entered/uploaded, the application can be certified and submitted in Sapphire. Certification is required by both the CIA and Administering Institution. Refer to Section 7.7 Certification and Submission of the guidelines for further details.

Before completing these steps:

- Review the application to ensure it is accurate and complete and meets all eligibility/application requirements.
  - Applicants retain responsibility for confirming that their application satisfies the stated eligibility requirements.
  - For funding schemes where the applicant has nominated a research budget, the summary tab automatically generates a summary of the requested budget from the relevant sections.
  - A checklist for applicants applying for NHMRC funding is provided at Section 8 of this Appendix of this document.
  - Ensure you have read and understood the assurances, acknowledgements and undertakings required of CIAs and Administering Institutions as part of this step. These are outlined in Section 7.7 - Certification and Submission of the TCR: Cultural, ethnic and linguistic diversity in dementia research grant opportunity guidelines.
  - Note that certification will lock down the application and prevent further editing. The final snapshot produced at this time will include relevant information from your 'My Profile'. Any subsequent changes to these areas of Sapphire will not appear in the application. If changes are needed after CIA certification but before submission to NHMRC, your RAO will need to reject the application in order for you to make the changes.
  - Note that your personal information may be provided to another Administering Institution for the purpose of certifying the application where a researcher is either currently receiving NHMRC funding or is on a different and separate application for NHMRC funding.

Instructions for certifying and submitting an application in Sapphire are provided in the *Sapphire Learning and Training Resources*.

Once submitted to NHMRC, your application will be considered final and no changes can be made unless the application is withdrawn for amendment before the closing date.

8. Checklist for applicants

Before creating an application:

- Ensure Sapphire Accounts for all CIs are active and mandatory 'My Profile' fields are complete (indicated by a red asterisk *).
- Familiarise yourself with the guidelines and *Sapphire Learning and Training*

Resources.

- Check closing date and time for application lodgement.
- Update your Sapphire 'My Profile' in accordance with requirements set out in this document.
- Read the relevant ethical guidelines/associated documentation if ethics approval is required for the proposed application.
- Inform your RAO of your intention to submit an application.
- Be aware of any Administering Institution internal deadlines and requirements for submission.

During the creation of an application:

- Check any minimum data requirements.
- Check eligibility requirements.
- Complete all parts of the application.
- Create and upload your Grant Proposal.
- Identify any Relative to Opportunity considerations, including Career Disruptions, where applicable, within your application.
- Consider any Aboriginal and Torres Strait Islander requirements for your application, including addressing any additional selection criteria.
- Make sure all required attachments are uploaded.

Before submitting an application:

- Read and understand the *Australian Code for the Responsible Conduct of Research, 2018*. Submission of an application indicates that the Administering Institution and research team understand and will comply with the principles and responsibilities set out in the Code.
- Check your compliance with formatting and page requirements.
- Ensure any Approvals or licences are acquired or applied for.
- Check all information is correct and complete.
- Familiarise yourself with your obligations should you be successful.
- Certify the application and ensure RAO certification and submission occur before the closing date and time.

Remember, your RAO is your primary contact for advice and assistance. RAOs will contact the Research Help Centre for further advice if required.
