# Supplementary material for: A culturally tailored iSupport model for dementia carers: Study protocol for a hybrid type I randomised controlled trial
Source: Int J Nurs Stud Adv. 2026 May 2;10:100546. doi: 10.1016/j.ijnsa.2026.100546 (PMC13136732; doi:10.1016/j.ijnsa.2026.100546)
Supplement: Supplementary file 2 [file mmc2.pdf]

OFFICIAL: Sensitive

Australian Government
National Health and Medical Research Council

NHMRC

SAPPHIRE

# Application Summary

| Application Details | |
|---|---|
| **Grant Opportunity:** | 2022 TCR Cultural Ethnic and Linguistic Diversity in Dementia Research |
| **Application ID:** | 2024551 |
| **Application Title:** | A ‘culturally tailored iSupport model’ for carers of people with dementia |
| **Chief Investigator A:** | Prof Lily Dongxia Xiao |
| **Administering Institution:** | Flinders University |
| **Grant Duration:** | 5 Years |

## Participating Institutions

| Participating Institution | Department | Research Effort (%) |
|---|---|---|
| Flinders University | College of Nursing & Health Sciences | 63 |
| Bolton Clarke | Bolton Clarke Research Institute | 8 |
| University of Wollongong | School of Nursing | 11 |
| Australian Nursing Home Foundation | Community aged care | 3 |
| Illawarra Shoalhaven Local Health District | Multicultural Health Service | 3 |
| Chinese Australian Services Society | Community aged care | 3 |
| Community Access and Services SA | Community aged care | 3 |
| The Society of Saint Hilarion | Community aged care | 3 |
| Greek Orthodox Community Of South Australia Incorporated | Community aged care | 3 |

## Research Team

| Role | Investigator | Primary Institution |
|---|---|---|
| CIA | Prof Lily Dongxia Xiao | Flinders University |
| CIB | Dr Rachel Milte | Flinders University |
| CIC | Dr Claudia Meyer | Bolton Clarke |
| CID | Dr Hui Chen Chang | University of Wollongong |
| CIE | Kham Tran | Flinders University |
| CIF | Dr Shahid Ullah | Flinders University |
| CIG | Assoc Prof Kate Laver | Flinders University |
| CIH | Ying Yu | Flinders University |
| CII | Prof Alison Kitson | Flinders University |

| Associate Investigator | Institution |
|---|---|
| Prof Lee-Fay Low | University of Sydney |
| Ms Helena Kyriazopoulos | The University of Adelaide |
| Dr Ron Sinclair | The University of Adelaide |
| Anne Margriet Pot | |
| Mr Mauricio Molinari Ulate | University of Salamanca |
| Dr Areti Efthymiou | |
| Dr Maddalena Fiordelli | University of Lugano |
| Agnieszka Chudecka | Multicultural Aged Care |
| Prof Henry Brodaty | University of New South Wales |

Mr Nikolaus Rittinghausen Centre for Cultural Diversity in Ageing

## Research Classification

| Broad Research Area |
| --- |
| Health Services Research |
| **Fields of Research** |
| HEALTH SCIENCES \| Nursing \| Aged care nursing |
| HEALTH SCIENCES \| Health services and systems \| Aged health care |
| ECONOMICS \| Applied economics \| Health economics |
| **Research Keywords** |
| dementia care - cultural perceptions - health service accessibility - translational research - health economics |

## Synopsis

Carers from culturally and linguistically diverse (CALD) groups experience structural discrimination in our health and social care systems. This barrier prevents carers from accessing and utilising care services for people living with dementia, attaining dementia care education and social support tailored to their culture and language. We propose a culturally tailored iSupport model (iSupport model) to mitigate these issues. The model includes bilingual and bicultural facilitator-enabled access to care services to meet the care needs of people with dementia, carer support groups and dementia care education for carers using iSupport program in carers' preferred language and carers' feedback on care services.

This study includes four study aims and collaborates with seven dementia care service providers. In phase 1, we will co-design strategies and co-create resources with stakeholders to implement the iSupport model using focus groups and interviews (aim 1). In phase 2, we will conduct a pragmatic, multicentre, type 1 hybrid effectiveness-implementation randomised controlled trial with 180 CALD carers from seven groups of various languages to determine the intervention effectiveness (aim 2), implementation strategies (aim3) and intervention cost-effectiveness (aim 4). Carers in the intervention group will receive the iSupport model intervention for 12 months. We will measure quality of life and health outcomes for carers and care recipients, the use of health and aged care services and the incremental cost per quality adjusted life years gained by the intervention compared to usual care. We will hold focus groups with carers and facilitators and analyse partner organisations' data to determine the implementation strategies.

This study is much needed because the iSupport model will improve health and quality of life for both carers and care recipients from CALD groups, improve cost-effectiveness of care services, and address structural discrimination in the care system.

## Media Summary

Our study aims to co-design and embed a culturally tailored iSupport model in dementia care services and evaluate its effectiveness with stakeholders. The model is designed to enhance culturally and linguistically congruent support for carers and improve their ability to manage dementia at home and relieve pressure on dementia care services. The expected outcomes are improved quality of life and health for carers and people with dementia, improved quality of care and cost-effective care.
