# Supplementary material for: A culturally tailored iSupport model for dementia carers: Study protocol for a hybrid type I randomised controlled trial
Source: Int J Nurs Stud Adv. 2026 May 2;10:100546. doi: 10.1016/j.ijnsa.2026.100546 (PMC13136732; doi:10.1016/j.ijnsa.2026.100546)
Supplement: Supplementary file 4 [file mmc4.pdf]

Australian Government
National Health and Medical Research Council

NHMRC

Application Assessment Summary

2022 TCR Cultural Ethnic and Linguistic Diversity in Dementia Research

Application ID: 2024551

Chief Investigator A: Professor Lily Dongxia Xiao

Administering Institution: Flinders University

Table 1: Summary of the assessment of your application against the 2022 TCR Cultural Ethnic and Linguistic Diversity in Dementia Research Assessment Criteria.

Criteria			
	1	Consumer and community involvement activities	5.750
	2	Support for consumer and community involvement	5.500
	3	Relevance and research impact for consumers and community	6.000
	4	Scientific quality and relevance to the objectives and expected outcomes of the proposed research	5.625
	5	Team capacity and record of achievement of the team in areas/disciplines relevant to this TCR – relative to opportunity	5.500
		**Overall Score**	**5.613**
