# Supplementary material for: A culturally tailored iSupport model for dementia carers: Study protocol for a hybrid type I randomised controlled trial
Source: Int J Nurs Stud Adv. 2026 May 2;10:100546. doi: 10.1016/j.ijnsa.2026.100546 (PMC13136732; doi:10.1016/j.ijnsa.2026.100546)
Supplement: Supplementary file 5 [file mmc5.docx]

**Information about trial registration data**

| **Data category** | **Information** |
| --- | --- |
| Primary registry and trial identifying number | Australia New Zealand Clinical Trials Registry https://anzctr.org.au/Trial/Registration/TrialReview.aspx?ACTRN=12625000587404, ACTRN12625000587404 |
| Date of registration in primary registry | 5 June 2025 |
| Source of monetary or material support | This project is funded by the 2022 National Health and Medical Research Council Targeted Call Research: Cultural, ethnic and linguistic diversity in dementia research (Grant ID: APP2024551) |
| Primary sponsor | National Health and Medical Research Council, Australian Government |
| Contact for public queries | Professor Lily XIAO; Email: [lily.xiao@flinders.edu.au](mailto:lily.xiao@flinders.edu.au) |
| Contact for scientific queries | Lily XIAO, PhD, Professor, Flinders University, Australia |
| Public title | Evaluation of a ‘culturally tailored iSupport model’ for carers of people with dementia |
| Scientific title | Impact of a ‘culturally tailored iSupport model’ on quality of life for carers of people with dementia |
| Settings of recruitment | Seven aged care organisations across three states (NSW, SA, VIC) in Australia |
| Health conditions or problems studied | Dementia care |
| Interventions | Interventions: This intervention titled 'culturally tailored iSupport model' includes facilitator-enabled carer psychoeducation, peer support, needs-based support and coaching and feedback on services, all delivered in carers’ preferred languages and cultural contexts. |
|  | Control: The trained facilitators will direct CALD carers in this group to Dementia Australia and iSupport program website for dementia care resources including the online iSupport manual in their preferred language using a flyer. |
| Key inclusion and exclusion criteria | Inclusion criteria: 1) Carers speak one of these languages at home: Italian, Greek, Mandarin, Cantonese, Vietnamese, Bahasa or Spanish; 2) Carers are aged 18 years or over; 3) Carers provide care for older people (aged greater than or equal to 65) living with dementia from a culturally and linguistically diverse background at least twice a week. If the care recipients have not been formally diagnosed with dementia but meet cognitive impairment using the “AD8 Dementia Screening Interview” with score equal or greater than 2. |
|  | Exclusion criteria: Carers will be excluded if they (1) have health conditions that may significantly impact their ability to participate in the study; (2) involve in other studies. |
| Study type | a pragmatic, multicentre, hybrid type 1 effectiveness–implementation randomised controlled trial |
| Date of first enrolment | 9 February 2026 |
| Target sample size | 150 (n = 75 in either the intervention or control group) |
| Recruitment status | Recruiting |
| Primary outcomes | Carers' quality of life using 12-Item Short-Form Health Survey (SF-12).  Care recipients’ Quality of Life in Alzheimer’s Disease (QOL-AD)-Proxy |
| Secondary outcomes | 1. Carers: Carers in the intervention group will be encouraged to provide feedback on the care services received by their care recipients. 2. Care recipients' Quality of Care Experience (QCE) Measure. 3. Carer recipients: Combined measure of hospital admissions, emergency department presentations, and use of permanent residential aged care. 4. Self-Administered Caregiving Self-Efficacy Scale (CSES-8) 5. The Revised Memory and Behaviour Problem Checklist 6. Facilitator experiences: Composite measure of facilitators’ perspectives on implementing and monitoring the model, including outcome evaluation, self-assessment, and observations on organisational knowledge and environmental adjustments during the implementation phase. 7. Carer recipients; Combined measure of health and social care visits (excluding MBS-covered services), collected using the Resource Utilisation in Dementia’ questionnaire. 8. Carers' Quality of Social Support Scale |
