# Supplementary material for: A culturally tailored iSupport model for dementia carers: Study protocol for a hybrid type I randomised controlled trial
Source: Int J Nurs Stud Adv. 2026 May 2;10:100546. doi: 10.1016/j.ijnsa.2026.100546 (PMC13136732; doi:10.1016/j.ijnsa.2026.100546)
Supplement: Supplementary file 6 [file mmc6.docx]

**Supplementary files**

**Supplementary file 1: Outline of the iSupport program and the culturally tailored case studies for dementia carers**

| iSupport Module | iSupport Units | Unfolding case studies | Related case studies |
| --- | --- | --- | --- |
| 1. Introduction to dementia | 1. What is dementia? | 1: Caring for a person with memory loss | 1.1: Lucia has forgotten to buy essential food  1.2: Care services enable Lucia to live at home  1.3: The care outcomes Isabella and Lucia have achieved |
| 2. Being a carer | 1. The journey together  2. Improving communication  3. Supported decision-making  4. Involving others | 2: Supporting the carer at the time of dementia diagnosis and beyond | 2.1: Challenges in supporting George to develop an advance care directive  2.2: Communication with George about his future care  2.3: Working with George to achieve the care outcomes  2.4: Challenges for George in managing his health conditions  2.5: Journey together to manage George’s health conditions  2.6: The care outcomes Althea and George have achieved  2.7: Challenges in managing hospital-to-home transitional care for George  2.8: Organising care services for George to smooth the transition  2.9: The care outcomes Althea has achieved |
| 3. Caring for yourself | 1. Reducing stress in everyday life  2. Making time for pleasant activities  3. Thinking differently | 3: Enabling carers to perform culturally tailored self-care | 3.1. Mei experiences stresses at the time of her husband’s dementia diagnosis  3.2. Culturally appropriate approach to relaxation activities  3.3: The self-care outcomes Mei has achieved  3.4: Mei experiences self-care challenges when her husband shows changes  3.5: Mei’s approach to coping with self-care challenges  3.6: Mei’s selfcare outcomes  3.7: Organising respite care in a care home for Li  3.8: Preparations for Li before respite care at a care home  3.9: The care outcomes Mei has achieved |
| 4. Providing everyday care | 1. Eating and drinking-more pleasant mealtimes  2. Eating, drinking and preventing health problems  3. Toileting and continence care  4. Personal care  5. An enjoyable day | 4: Supporting carers in the late stage of dementia care | 4.1: Challenges Nam experiences  4.2: Aged care services Nam used to cope with the challenges  4.3: The care outcomes Nam has achieved  4.4: Challenges for Nam in managing his father’s pressure sores and other health issues  4.5: Various care services Nam used to cope with the challenges  4.6: The care outcomes Nam has achieved  4.7: Challenges for Nam in providing palliative care for his father  4.8: Palliative care services Nam used  4.9: The care outcomes Nam has achieved |
| 5. Dealing with changed behaviour | 1. Introduction to behaviour changes  2. Memory loss  3. Aggression  4. Depression, anxiety and apathy  5. Difficulty sleeping  6. Delusions and hallucinations  7. Repetitive behaviour  8. Walking and getting lost  9. Changes in judgement  10. Putting it all together | 5: Enabling carers to care for their loved ones with changed behaviours | 5.1: Enabling carers to care for their loved ones with aggressive behaviours  5.1.1: Challenges Mrs Rodríguez experiences  5.1.2: Using the ABC approach to understand aggressive behaviour  5.1.3: The care outcomes Mrs Rodríguez has achieved  5.2: Enabling carers to care for their loved ones with apathy  5.2.1: Challenges Dewi experiences  5.2.2: Using a person-centred care approach to care for the person with apathy  5.2.3: The care outcomes Dewi has achieved  5.3: Enabling carers to care for their loved ones with hallucination  5.3.1: Challenges Lan experiences  5.3.2: Using the ABC approach to understand hallucination  5.3.3: The care outcomes Lan has achieved  5.4: Enabling carers to care for their loved ones with wandering  5.4.1: Challenges Bella experiences  5.4.2: Using the ABC approach to understand wandering  5.4.3: The care outcomes Bella has achieved  5.5: Enabling carers to care for their loved ones with depressive symptoms  5.5.1: Challenges Liang experiences  5.5.2: Using the ABC approach to understand depressive symptoms  5.5.3: The care outcomes Liang has achieved  5.6: Enabling carers to care for their loved ones with toileting issues  5.6.1: Challenges Maria experiences  5.6.2: Using the ABC approach to understand toileting issues  5.6.3: The care outcomes Maria has achieved  5.7: Enabling carers to care for their loved ones with repetitive behaviours  5.7.1: Challenges Mr Zhen experiences  5.7.2: Using the ABC approach to understand repetitive behaviours  5.7.3: The care outcomes Mr Zhen has achieved  5.8: Enabling carers to care for their loved ones with sleep issues  5.8.1: Challenges Mrs. Romano experiences  5.8.2: Using the ABC approach to understand sleep issues  5.8.3: The care outcomes Mrs. Romano has achieved |
| 6. Consumer-directed aged care and dementia care | 1. Making informed choices in dementia care  2. Services that are available for younger onset dementia  3. Carer support  4. Dementia Behaviour Management Advisory Service  5. Support at Home  6. Relinquishing the carer role | 6: Enabling carers to access care services | 6.1: Enabling carers to access aged care services  6.1.1: Challenges and opportunities for Mr Chen to access My Aged Care for his wife  6.2: Enabling carers to relinquish their carer role  6.2.1: Challenges for Maria in relinquishing her carer’s role |

ABC= Antecedent, Behaviour, and Consequences.

**Supplementary file 2: Carer coaching plan**

My first name: _________ My facilitator’s first name: ______________ Date for the plan: _________ The learning module or unit(s) related to this plan:

| **Items** | **Please fill in this column** |
| --- | --- |
| Problems I encounter are: |  |
| My coaching goals for resolving the problems are: |  |
| The advice that I have received from my facilitator and/or my peers: |  |
| Actions I have taken are: |  |
| Outcomes of the coaching regarding my selfcare: |  |
| Outcomes of the coaching regarding the care of my care recipient: |  |

**Supplementary file 3: Facilitator coaching tool**

Carer’s first name:______________ Organisation’s name:____________

Facilitator’s first name:_______________ Coaching date: ________________

| **Five coaching steps** | **Discussion points** | **Facilitator records** (date started and completed; any issues identified and resolutions) |
| --- | --- | --- |
| Step 1: Needs assessment | Select a suitable need assessment tool for the carer to undertake the needs assessment. |  |
| Step 2: Goal setting | Enable the carer to set out realistic and achievable selfcare goals. |  |
|  | Enable carers to set out realistic and achievable care goals for the care recipient. |  |
| Step 3: Skill training | Engage the carer in dementia care skill training using relevant learning modules or units from the iSupport manual or other education resources. |  |
| Step 4: Needs-based one-on-one coaching or group coaching | Encourage the carer to book one-on-one coaching sessions to discuss problems they encounter and strategies to resolve the problems. |  |
|  | Organise group coaching activities in peer support group meetings to enable experienced carers to coach less experienced carers when appropriate. |  |
| Step 5: Evaluation | Enable the carer to evaluate whether they have achieved the goals and plan for other coaching sessions when needed. |  |
