# Supplementary material for: A culturally tailored iSupport model for dementia carers: Study protocol for a hybrid type I randomised controlled trial
Source: Int J Nurs Stud Adv. 2026 May 2;10:100546. doi: 10.1016/j.ijnsa.2026.100546 (PMC13136732; doi:10.1016/j.ijnsa.2026.100546)
Supplement: Supplementary file 3 [file mmc3.pdf]

Australian Government
National Health and Medical Research Council

NHMRC

In reply, please quote: NHMRC 2022 TCR Cultural Ethnic and Linguistic Diversity in Dementia Research Application 2024551

Professor Lily Dongxia Xiao

lily.xiao@flinders.edu.au

********* UNDER EMBARGO AND PROVIDED IN CONFIDENCE *********

This advice and document/s referred to below are provided under strict embargo and as such, on an in confidence basis. **The document/s and the information are not to be made public at this time by institutions or recipients**. NHMRC will notify your Administering Institution when your outcome is no longer under embargo.

****

Dear Professor Xiao

Application ID: 2024551
Grant Opportunity: 2022 TCR Cultural Ethnic and Linguistic Diversity in Dementia Research
Application Title: A 'culturally tailored iSupport model' for carers of people with dementia

I am pleased to advise that the Minister for Health and Aged Care has approved your application (2024551) for National Health and Medical Research Council (NHMRC) 2022 TCR Cultural Ethnic and Linguistic Diversity in Dementia Research - commencing in 2023.

This letter provides you with important information about the offer of Funding made to Flinders University for this application.

Assessment Details

Where available[1], information about the assessment of your application is provided in a separate Application Assessment Summary.

Accepting this offer

The offer of Funding for your Application is made under NHMRC's Funding Agreement (the Funding Agreement) between the Australian Government and your Administering Institution. Your Administering Institution is responsible for informing you about the requirements of the Funding Agreement (including its Schedules), the Direct Research Costs guidelines and other applicable policies[2].

Your Administering Institution has until 15/08/2023 to certify that the information required prior to payment being made (see below) has been entered into NHMRC's Grant Management System, and to advise NHMRC of its acceptance of the offer. If the offer is not

[1] An Application Assessment Summary is not available for applications to grant opportunities where NHMRC does not perform the peer review.

[2] Funding Agreement, Direct Research Costs Guidelines and other policies are available on the webpage at: (https://nhmrc.gov.au/funding/manage-your-funding/funding-agreement).

accepted by this date it may lapse. If you wish to discuss this offer of Funding, or have any queries, please contact your Research Administration Officer (RAO).

Information required prior to payment being made

Where applicable, and except where otherwise indicated, NHMRC will temporarily withhold some or all of the Funding under subclause 15.2.a of the Funding Agreement with your Administering Institution until Specified Personnel with outstanding obligations from previous NHMRC grants, including submission of a Final Report, have met those obligations.

In some circumstances, CIAs may need to provide additional ethics information. This information must be entered into NHMRC's Grant Management System by the CIA and certified by the RAO. Should you have any questions concerning the provision of such information, please speak to your RAO.

If you need to seek approval to defer the start date of this grant, please refer to the Grantee Variations webpage or speak with your RAO.

Funding

As set out in the Schedule to the Funding Agreement, the 2022 TCR Cultural Ethnic and Linguistic Diversity in Dementia Research 2024551, has been awarded $1,555,078.05. Where applicable, this budget has been assessed by the peer review panel as sufficient to complete the aims and objectives of the research proposal stated in the application for funding. Any conditions relevant to receiving the Funding are set out in the Schedule to the Funding Agreement and, where applicable, the associated Funding Policy. All expenditure must be in accordance with the requirements of the Funding Agreement.

Participation in NHMRC Peer Review

NHMRC relies on the ongoing participation of the research community to ensure that every application receives expert peer review. NHMRC is grateful for this contribution which is acknowledged on its website's peer review honour roll.

To ensure that applications for future rounds are appropriately assessed, all Specified Personnel working on NHMRC Funded Research Activities are reminded that they may be requested to make themselves available to contribute to the peer review process, in accordance with clause 23.1 of the Funding Agreement.

Accordingly, we ask that you ensure your CV/Profile information is up to date in Sapphire to assist in the identification of appropriate peer reviewers.

Yours sincerely

[Authorised for electronic transmission]

Marita Sloan
A/g Executive Director
Research Quality & Priorities Branch
